# Betulinic Acid Decorated with Polar Groups and Blue Emitting BODIPY Dye: Synthesis, Cytotoxicity, Cell-Cycle Analysis and Anti-HIV Profiling

**DOI:** 10.3390/biomedicines9091104

**Published:** 2021-08-28

**Authors:** David Kodr, Jarmila Stanková, Michaela Rumlová, Petr Džubák, Jiří Řehulka, Tomáš Zimmermann, Ivana Křížová, Soňa Gurská, Marián Hajdúch, Pavel B. Drašar, Michal Jurášek

**Affiliations:** 1 Department of Chemistry of Natural Compounds, University of Chemistry and Technology Prague, 16628 Prague, Czech Republic; david.kodr@vscht.cz (D.K.); tomas.zimmermann@vscht.cz (T.Z.); pavel.drasar@vscht.cz (P.B.D.); 2 Institute of Molecular and Translational Medicine, Faculty of Medicine and Dentistry, Palacký University and University Hospital in Olomouc, 77900 Olomouc, Czech Republic; jarmila.stankova@upol.cz (J.S.); petr.dzubak@upol.cz (P.D.); jiri.rehulka@upol.cz (J.Ř.); sona.gurska@upol.cz (S.G.); marian.hajduch@upol.cz (M.H.); 3 Department of Biotechnology, University of Chemistry and Technology Prague, 16628 Prague, Czech Republic; michaela.rumlova@vscht.cz (M.R.); ivana.krizova@vscht.cz (I.K.)

**Keywords:** betulinic acid, BODIPY, bevirimat, cytotoxicity, cancer, cell-cycle, fluorescent microscopy, maturation inhibitor

## Abstract

Betulinic acid (BA) is a potent triterpene, which has shown promising potential in cancer and HIV-1 treatment. Here, we report a synthesis and biological evaluation of 17 new compounds, including BODIPY labelled analogues derived from BA. The analogues terminated by amino moiety showed increased cytotoxicity (e.g., BA had on CCRF-CEM IC_50_ > 50 μM, amine **3** IC_50_ 0.21 and amine **14** IC_50_ 0.29). The cell-cycle arrest was evaluated and did not show general features for all the tested compounds. A fluorescence microscopy study of six derivatives revealed that only **4** and **6** were detected in living cells. These compounds were colocalized with the endoplasmic reticulum and mitochondria, indicating possible targets in these organelles. The study of anti-HIV-1 activity showed that **8**, **10**, **16**, **17** and **18** have had IC_50i_ > 10 μM. Only completely processed p24 CA was identified in the viruses formed in the presence of compounds **4** and **12**. In the cases of **2**, **8**, **9**, **10**, **16**, **17** and **18**, we identified not fully processed p24 CA and p25 CA-SP1 protein. This observation suggests a similar mechanism of inhibition as described for bevirimat.

## 1. Introduction

**Figure 1:**
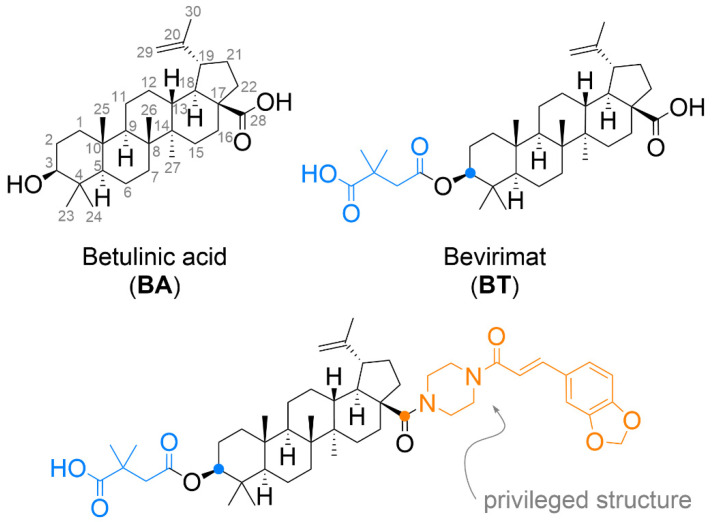
Chemical structure of betulinic acid and its derivatives.

Betulinic acid (BA) is a natural pentacyclic triterpene of the lupane type (Figure 1). Despite its low solubility in aqueous solutions, this substance is gaining attention with its wide range of interesting biological activity. BA is often derivatized to increase solubility, enhance the therapeutic effect, and target the drug to the specific site of action [1]. BA shows a significant degree of selectivity for cytotoxicity against a variety of tumour cells [2,3,4] and activity against HIV-1 [5]. There are several possible mechanisms of action of BA (reviewed in [6]), which provide an advantage in the development of resistance to one of the mechanisms and may thus find application in the treatment of tumours resistant to current chemotherapeutics [6]. One is the direct action of BA on the mitochondrial membrane, leading to an increase of outer membrane permeability, its depolarization and release of cytochrome *c* into the cytosol. It is then responsible for triggering apoptosis [7]. Among other effects of BA, reactive oxygen species can be formed causing non-specific damage to mitochondria [8,9], followed by the induction of caspase activity [10]. BA exhibits topoisomerase I28 inhibitory activity, and through the proteasome-dependent independent regulatory pathway, is responsible for the function of the transcription factors Sp1, Sp3 and Sp4 inhibition [11]. It is also able to inhibit the activation of the stress transcription factor NF-κB [12]. A slightly different way in which tumour growth is inhibited is a complete or partial slowing of angiogenesis [13]. Later studies have shown that the antiangiogenic effect is achieved via modulation of mitochondria [14].

BA has been shown to have anti-HIV-1 activity in the past. Although the test results were not groundbreaking, and the effect was observed only at relatively high concentrations [5] This discovery inevitably led to the synthesis of several other analogues. One of the derivatives with strong anti-HIV-1 activity was 3-*O*-(3,3-dimethylsuccinyl) betulinic acid, known as bevirimat (Figure 1, BT) [15]. BT acts as an inhibitor of HIV-1 particle maturation. Inhibition of viral particle maturation appears to be a critical point of therapeutic intervention. During the maturation phase, the viral protease cleaves the Gag polyprotein while releasing the individual structural proteins. The final step is the cleavage of p25 CA-SP1 to a functional p24 CA protein. Inhibition of the last step of maturation results in virus particles with aberrantly formed mature cores that are incapable of further infection [16]. BT advanced to the second phase of clinical testing [17,18,19], during which virus reduction was observed in only 40–50% of patients. The remainder of the patients developed resistance due to natural polymorphic variation in the Gag polyprotein [20]. With this result, the clinical studies were terminated.

Given the important features of BA mentioned above, it is no surprise that many research groups addressed it. Hundreds of derivatives have been prepared over the last few decades. However, with derivatization, for example, the expected effect disappeared, resistance developed rapidly, or toxicity to normal cells increased dramatically. For anti-HIV derivatives, several so-called “privileged structures” (Figure 1), structural motifs that can be the basis for the design of an effective drug, were found [21,22]. BA is most often chemically modified at C-3 and C-28 positions. Addition to the double bond between carbon atoms C-20 and C-30 usually does not significantly enhance activity, on the contrary, the activity often disappears. This finding generally applies to both anti-cancer and anti-HIV effects [23,24,25]. Recent works have confirmed that the presence of an extra amine group introduced by conjugation into a BA molecule can significantly increase antitumour potency [26,27].

This work presents the preparation and biological evaluation of new analogues of BA and BT containing an amino group. In the past, fluorescent analogues of BA labelled with green-emitting BODIPY (4,4-difluoro-4-bora-3a,4a-diaza-*s*-indacene) [28,29] and red-emitting Rhodamine B [30] were synthesized to study its localization and trafficking in living cells. In this work, we synthesized and studied new derivatives of BA and BT labelled at C-3 and C-28 positions using a small blue-emitting BODIPY dye.

## 2. Materials and Methods

### 2.1. Chemical Synthesis

Aluminium silica gel sheets for detection in UV light (TLC Silica gel 60 F254, Merck, Darmstadt, Denmark) were used for thin-layer chromatography (TLC), subsequent visualization was proceeded by a diluted solution of sulfuric acid in methanol and plates were heated. Silica gel (30–60 μm, SiliTech, MP Biomedicals, Costa Mesa, CA, USA) was used for column chromatography. NMR Spectra were recorded by Agilent-MR DDR2 (Santa Clara, CA, USA). HRMS were measured by LTQ ORBITRAP VELOS with HESI+/HESI- ionization (Thermo Scientific, Waltham, MA, USA). For microwave synthesis, an Initiator Classic 355,301 (Biotage, Uppsala, Sweden) was used.

The following chemicals were purchased from TCI Europe (Zwijndrecht, Belgium): *N*,*N*,*N*-triethylamine—Et_3_N (>99%), 4-dimethylaminopyridine—4-DMAP (>99%), 1-(3-dimethylaminopropyl)-3-ethylcarbodiimide hydrochloride—EDCI (>98%), *N*,*N′*-dicyclohexylcarbodiimide—DCC (>98%), 1-hydroxybenzotriazole monohydrate—HOBt (>97%), triphenylphosphine—PPh_3_ (>95%), *p*-toluenesulfonic acid monohydrate—*p*-TsOH (>98%), and palladium on carbon—Pd/C (10%). The following chemicals were purchased from Sigma-Aldrich (Prague, Czech Republic): 3-azidopropylamine (≥95%), 1-(2-*N*-boc-aminoethyl)piperazine (≥95%), β-alanine (99%). Betulinic acid (BA) was purchased from Betulinines (Stříbrná skalice, Czech Republic).

The solvents for column chromatography and reactions were purchased from PENTA (Praha, Czech Republic) and were used without further distillation.

#### Compound Synthesis and Characterization

8-*N*-(3-Azidopropyl)amino-4,4-difluoro-4-bora-3a,4a-diaza-*s*-indacene (**BODIPY-N_3_**)

To a solution of **BODIPY-SMe** (205 mg, 0.86 mmol) in DCM (10 mL), 3-azidopropylamine (95 mg, 0.95 mmol) was added and the mixture was stirred for 30 min at RT. The solvents were evaporated under reduced pressure and the residue was taken up with AcOEt and the product was precipitated by the addition of hexanes. **BODIPY-N_3_** (243 mg, 0.83 mmol) was obtained as a yellowish solid in 97% yield. R_F_ = 0.55 in hexanes-AcOEt 1:1. ^1^H NMR (400 MHz, CD3OD) δ ppm: 2.11 (quin, J = 6.7 Hz, 2 H), 3.56 (t, J = 6.5 Hz, 2 H), 3.87 (t, J = 7.4 Hz, 2 H), 6.39 (br. s., 1 H), 6.55 (br. s, 1 H), 7.32 (br. s, 1 H), 7.36 (br. s., 2 H), 7.57 (s, 1 H). ^13^C NMR (101 MHz, CD_3_OD) δ ppm: 27.00, 44.08, 48.76, 112.67, 113.99, 115.78, 123.16, 130.78, 133.76, 148.87. HRMS-ESI: calculated 290.12628 Da, found *m*/*z* 291.13312 [M+H]^+^.

8-*N*-(3-Aminopropyl)amino-4,4-difluoro-4-bora-3a,4a-diaza-*s*-indacene (**BODIPY-NH_2_**)

To a solution of **BODIPY-N_3_** (150 mg, 0.52 mmol) in AcOEt (8 mL), was added Pd/C (80 mg) and the mixture was stirred under hydrogen atmosphere for 2 h. The catalyst was filtered off and the solvents were evaporated under reduced pressure. The residue was taken up with AcOEt and the product was obtained after precipitation with hexane. **BODIPY-NH_2_** (96 mg, 0.36 mmol) was obtained as yellow solid in 70% yield. R_F_ = 0.15 in DCM-MeOH 20:1 (*v*/*v*). ^1^H NMR (400 MHz, CD_3_OD) δ ppm: 1.98 (quin, J = 6.7 Hz, 2 H), 2.87 (t, J = 6.7 Hz, 2 H), 3.83 (t, J = 7.0 Hz, 2 H), 6.37 (br. s, 1 H), 6.52 (br. s, 1 H), 7.27 (br. s, 1 H), 7.30–7.36 (m, 2 H), 7.55 (br. s, 1 H). ^13^C NMR (101 MHz, CD_3_OD) δ ppm: 29.67, 39.12, 45.50, 112.53, 113.78, 115.51, 123.15, 130.40, 133.46, 148.73. HRMS-ESI: calculated 264.13578 Da, found *m*/*z* 263.12810 [M-H]^−^.

8-*N*-(β-Alanyl)amino-4,4-difluoro-4-bora-3a,4a-diaza-*s*-indacene (**BODIPY-CO_2_H**)

To a solution of **BODIPY-SMe** (220 mg, 0.92 mmol) in DMSO (5 mL), was added a solution of β-Ala (91 mg, 1.02 mmol) in H_2_O (2 mL). The mixture was stirred at 30 °C for 16 h. The solvents were removed under reduced pressure and the residue was diluted with CHCl_3_ (100 mL). The product was precipitated by the addition of cyclohexane. **BODIPY-CO_2_H** (175 mg, 0.63 mmol) was obtained as yellow solids in 68% yield. R_F_ = 0.38 in hexanes-AcOEt 1:1. ^1^H NMR (400 MHz, CD_3_OD) δ ppm: 2.87 (t, J = 6.9 Hz, 2 H), 4.00 (t, J = 6.9 Hz, 2 H), 6.35 (br. s., 1 H), 6.53 (br. s., 1 H), 7.27–7.31 (m, 1 H), 7.33 (br. s., 2 H), 7.55 (br. s., 1 H). ^13^C NMR (101 MHz, CD_3_OD) δ ppm: 31.71, 42.48, 112.67, 114.02, 115.89, 123.26, 130.78, 133.83, 148.75, 173.03. HRMS-ESI: calculated 279.09906 Da, found *m*/*z* 278.09139 [M-H]^−^.

(3β)-*N*-(3-Azidopropyl)-3-hydroxylup-20(29)-ene-28-amide (**1**)

To a solution of BA (200 mg, 0.44 mmol) and 4-DMAP (59 mg, 0.48 mmol) in DMF (3 mL), 3-azidopropylamine (53 mg, 0.53 mmol), HOBt (65 mg, 0.48 mmol) and EDCI (93 mg, 0.48 mmol) were sequentially added. The mixture was stirred at RT for 36 h. Solvents were removed under reduced pressure and the residue was chromatographed twice (i. DCM-MeOH 100:1, *v/v* ii. DCM→DCM-MeOH 70:1, *v*/*v*). Azide **1** (200 mg, 0.37 mmol) was obtained as white solids in 84% yield. R_F_ = 0.48 in DCM-MeOH 40:1 (*v*/*v*). ^1^H NMR (400 MHz, CDCl_3_) δ ppm: 0.63–0.70 (m, 1 H), 0.75 (s, 3 H), 0.81 (s, 3 H), 0.83–0.91 (m, 1 H), 0.93 (s, 3 H), 0.96 (s, 3 H), 0.96 (s, 3 H), 0.97–1.05 (m, 1 H), 1.12–1.65 (m, 19 H), 1.67 (s, 3 H), 1.69–1.72 (m, 1 H), 1.75–1.82 (m, 2 H), 1.90–1.95 (m, 1 H), 2.38–2.48 (m, 1 H), 3.08 3.20 (m, 2 H), 3.32 (s, 4 H), 4.58 (s, 1 H), 4.73 (s, 1 H), 5.85 (t, J = 5.9 Hz, 1 H); Figure S1. ^13^C NMR (101 MHz, CDCl_3_) δ ppm: 14.29, 15.01, 15.79, 15.80, 17.93, 19.13, 20.57, 25.26, 27.05, 27.63, 28.68, 29.10, 30.51, 33.40, 34.04, 36.61, 36.84, 37.40, 38.05, 38.36, 38.49, 40.40, 42.11, 46.38, 49.31, 49.74, 50.27, 55.02, 55.30, 78.58, 109.01, 150.50, 175.96; Figure S3. HRMS-ESI: calculated 538.42468 Da, found *m*/*z* 561.41394 [M+Na]^+^; Figure S2.

4-{[(3β)-28-[(3-Azidopropyl)amino]-28-oxolup-20(29)-ene-3-yl]oxy}-2,2-dimethyl-4-oxo-butanoic acid (**2**)

To a solution of **1** (200 mg, 0.37 mmol) and 4-DMAP (73 mg, 0.59 mmol) in THF (2 mL), 2,2-dimethylsucccinic anhydride (238 mg, 1.86 mmol) and *p*-TsOH were added and the mixture was stirred for 2 h at 130 °C in microwave reactor (MW). The mixture was diluted with H_2_O (20 mL) and extracted with DCM (4 × 15 mL). Combined organic layers were dried over Na_2_SO_4_, filtered and the solvents were evaporated under reduced pressure. The residue was chromatographed two times (i. DCM-MeOH 100:1, *v*/*v*; ii. hexanes-AcOEt 3:1, *v*/*v*). Compound **2** (94 mg, 0.14 mmol) was obtained as white solids in 38% yield. R_F_ = 0.60 in hexanes-AcOEt, 1:1. ^1^H NMR (400 MHz, CDCl_3_) δ ppm: 0.73–0.76 (m, 1 H), 0.79 (s, 3 H), 0.82 (s, 6 H), 0.92 (s, 3 H), 0.95 (s, 3 H), 0.97–1.01 (m, 1 H), 1.12 1.17 (m, 1 H), 1.27 (s, 3 H), 1.29 (s, 3 H), 1.30–1.67 (m, 17 H), 1.67 (s, 3 H), 1.71 (d, J = 7.0 Hz, 1 H), 1.75–1.81 (m, 2 H), 1.90–1.93 (m, 1 H), 2.39–2.46 (m, 1 H), 2.52–2.68 (m, 2 H), 3.08 3.14 (m, 1 H), 3.22–3.33 (m, 2 H), 3.33–3.43 (m, 4 H), 4.45–4.50 (m, 1 H), 4.58 (s, 1 H), 4.73 (s, 1 H), 5.86 (t, J = 5.9 Hz, 1 H); Figure S4. ^13^C NMR (101 MHz, CDCl3) δ ppm: 14.60, 16.14, 16.46, 18.13, 19.44, 20.92, 23.59, 24.99, 25.27, 25.55, 25.58, 27.88, 29.02, 29.42, 30.83, 33.73, 34.30, 36.97, 37.08, 37.70, 37.72, 38.40, 40.44, 40.76, 42.45, 44.69, 46.74, 49.65, 50.06, 50.50, 55.47, 55.65, 81.51, 109.42, 109.99, 150.81, 170.95, 176.38, 182.48; Figure S5. HRMS-ESI: calculated 666.47202 Da, found *m*/*z* 667.47921 [M+H]^+^, 689.46125 [M+Na]^+^ and 705.43463 [M+K]^+^; Figure S6.

(3β)-*N*-(3-Aminopropyl)-3-hydroxylup-20(29)-ene-28-amide (**3**) [31]

A solution of **2** (339 mg, 0.63 mmol) and PPh_3_ (248 mg, 0.95 mmol) in THF (10 mL) was stirred for 3h at RT. Water (1 mL) was added and the mixture was stirred for additional 20 h at RT. Solvents were evaporated under reduced pressure and the residue was chromatographed (CHCl_3_-MeOH 20:1, *v/v* + 0.5% Et_3_N → 10:1, *v/v* + 0.5% Et_3_N). Compound **3** (278 mg, 0.54 mmol) was obtained as white solids in 86% yield. R_F_ = 0.15 in DCM-MeOH 10:1 (*v*/*v*) + 0.5% Et_3_N. ^1^H NMR (400 MHz, CD_3_OD) δ ppm: 0.70–0.75 (m, 1 H), 0.77 (s, 3 H), 0.88 (s, 3 H), 0.91–0.96 (m, 1 H), 0.97 (s, 3 H), 0.99 (s, 3 H), 1.02 (s, 3 H), 1.04–1.10 (m, 1 H), 1.15–1.69 (m, 20 H), 1.71 (s, 3 H), 1.80–1.95 (m, 2 H), 2.10–2.19 (m, 1 H), 2.55–2.64 (m, 1 H), 2.69 (t, J = 6.9 Hz, 2 H), 3.07–3.18 (m, 2 H), 3.20–3.32 (m, 2 H), 4.60 (s, 1 H), 4.72 (s, 1 H); Figure S7. ^13^C NMR (101 MHz, CD_3_OD) δ ppm: 13.80, 14.78, 15.46, 15.49, 18.07, 18.37, 20.78, 25.58, 26.64, 27.28, 29.22, 30.57, 32.01, 32.75, 34.24, 35.77, 36.95, 37.53, 38.11, 38.25, 38.57, 38.72, 40.61, 42.14, 46.67, 50.00, 50.68, 55.49, 55.57, 78.26, 108.63, 150.86, 177.88; Figure S8. HRMS-ESI: calculated 512.43418 Da, found *m*/*z* 513.44206 [M+H]^+^; Figure S9.

(3β)-*N*-[*N’*-(4,4-Difluoro-4-bora-3a,4a-diaza-*s*-indacene-8-yl)-3-aminopropyl]-3-hydroxylup-20(29)-ene-28-amide (**4**)

To a solution of BA (50 mg, 0.11 mmol) and **BODIPY-NH_2_** (32 mg, 0.12 mmol) in DMF (3 mL), 4-DMAP (15 mg, 0.12 mmol), HOBt (16 mg, 0.12 mmol) and EDCI (23 mg, 0.12 mmol) were added. The mixture was stirred for 20h at RT. The solvents were evaporated under reduced pressure and residue was chromatographed (hexanes-AcOEt 1:1). Compound **4** (56 mg, 0.08 mmol) was obtained as yellow-green solid in 73% yield. R_F_ = 0.27 in hexanes-AcOEt 1:1. ^1^H NMR (400 MHz, CDCl_3_) δ ppm: 0.66–0.70 (m, 1 H), 0.74 (s, 3 H), 0.80 (s, 3 H), 0.84–0.89 (m, 1 H), 0.90 (s, 3 H), 0.96 (s, 3 H), 1.00 (s, 3 H), 1.01–1.12 (m, 1 H), 1.17–1.71 (m, 20 H), 1.72 (s, 3 H), 1.73–1.80 (m, 2 H), 1.87–2.04 (m, 4 H), 2.48 2.55 (m, 1 H), 3.14–3.21 (m, 2 H), 3.28–3.36 (m, 2 H), 3.73 (q, J = 5.5 Hz, 2 H), 4.65 (s, 1 H), 4.77 (s, 1 H), 6.27 (t, J = 5.9 Hz, 1 H), 6.45 (br. s., 1 H), 6.50 (br. s., 1 H), 7.12 (br. s, 1 H), 7.51 (br. s., 1 H), 7.67 (br. s., 2 H), 9.73 (t, J = 5.3 Hz, 1 H); Figure S10. ^13^C NMR (101 MHz, CDCl_3_) δ ppm: 14.67, 15.35, 16.12, 16.15, 18.25, 19.46, 20.96, 25.61, 27.38, 27.96, 29.15, 29.53, 30.88, 33.53, 34.34, 36.75, 37.17, 37.94, 38.48, 38.72, 38.84, 40.81, 42.48, 44.48, 46.90, 50.10, 50.58, 55.35, 55.72, 78.95, 109.78, 113.37, 114.28, 116.74, 122.42, 125.66, 131.71, 134.28, 134.30, 149.07, 150.40, 179.08; Figure S11. HRMS-ESI: calculated 702.48556 Da, found *m*/*z* 725.47504 [M+Na]+ and 741.44867 [M+K]+; Figure S12.

(3β)-*N*-(3-Azidopropyl)-3-[*N´*-(4,4-difluoro-4-bora-3a,4a-diaza-*s*-indacene-8-yl)-β-alanyl]-oxy-lup-20(29)-ene-28-amide (**5**)

To a solution of **1** (130 mg, 0.24 mmol) and 4-DMAP (59 mg, 0.48 mmol) in dry DCM (5 mL), **BODIPY-CO_2_H** (101 mg, 0.36 mmol) and DCC (100 mg, 0.48 mmol) were added. The mixture was stirred at RT for 16 h. DCU was filtered off and the solvents were removed under reduced pressure. The crude was chromatographed (DCM-MeOH 100:1, *v*/*v*), and the material thus obtained was dissolved in AcOEt and precipitated by the addition of hexanes and chromatographed once again (DCM-MeOH 100:1, *v*/*v*) to obtain pure **5** (160 mg, 0.20 mmol) as yellow solid in 83% yield. R_F_ = 0.62 in DCM-MeOH 40:1 (*v*/*v*). ^1^H NMR (400 MHz, CDCl_3_) δ ppm: 0.78–0.82 (m, 1 H), 0.85 (s, 3 H), 0.85 (s, 6 H), 0.94 (s, 3 H), 0.97 (s, 3 H), 0.99–1.05 (m, 2 H), 1.14–1.18 (m, 1 H), 1.22–1.66 (m, 17 H), 1.69 (s, 3 H), 1.72 (m, 1 H), 1.75–1.81 (m, 2 H), 1.89–1.97 (m, 1 H), 2.42–2.50 (m, 1 H), 2.80 (t, J = 6.3 Hz, 2 H), 3.09–3.16 (m, 1 H), 3.23–3.39 (m, 4 H), 3.94 (q, J = 6.1 Hz, 2 H), 4.56–4.60 (m, 1 H), 4.60 (s, 1 H), 4.74 (s, 1 H), 5.83 (t, J = 5.9 Hz, 1 H), 6.44 (br. s., 2 H), 7.01 (br. s., 2 H), 7.45–7.68 (m, 2 H), 7.73 (t, J = 5.3 Hz, 1 H); Figure S13. ^13^C NMR (101 MHz, CDCl_3_) δ ppm: 14.59, 16.17, 16.19, 16.54, 18.14, 19.47, 20.96, 23.73, 25.54, 28.06, 29.04, 29.43, 30.85, 32.63, 33.74, 34.27, 36.98, 37.11, 37.70, 37.87, 38.33, 38.39, 40.77, 42.48, 42.53, 46.74, 55.42, 55.65, 76.70, 77.02, 77.34, 82.86, 109.44, 114.00, 114.79, 115.12, 123.08, 132.60, 135.52, 147.98, 150.82, 171.54, 176.32; Figure S14. HRMS-ESI: calculated 799.51318 Da, found *m*/*z* 822.50287 [M+Na]^+^ and 838.47620 [M+K]^+^; Figure S15.

(3β)-*N*-(3-Aminopropyl)-3-[*N*´-(4,4-difluoro-4-bora-3a,4a-diaza-s-indacene-8-yl)-β-alanyl]-oxy-lup-20(29)-ene-28-amide (**6**)

To a solution of **5** (60 mg, 0.08 mmol) in dry THF (3 mL), PPh_3_ (26 mg, 0.10 mmol) was added. After stirring for 3h, H_2_O was added and the mixture was stirred for 20 h. Solvents were removed under reduced pressure and the product was chromatographed (DCM-MeOH 9:1, *v/v* → MeOH-H_2_O 100:1, *v*/*v*) to obtain **6** (25 mg, 0.03 mmol) as yellow solids in 43% yield. R_F_ = 0.12 in DCM-MeOH 10:1 (*v*/*v*) + 0.5% Et_3_N. ^1^H NMR (400 MHz, CDCl_3_) δ ppm: 0.79–0.82 (m, 1 H), 0.85 (s, 3 H), 0.85 (s, 6 H), 0.95 (s, 3 H), 0.97 (s, 3 H), 0.98–1.03 (m, 2 H), 1.12–1.16 (m, 1 H), 1.26–1.65 (m, 22 H), 1.69 (s, 3 H), 1.72–1.77 (m, 2 H), 1.92–1.98 (m, 1 H), 2.45–2.51 (m, 1 H), 2.77–2.85 (m, 4 H), 3.11–3.18 (m, 1 H), 3.30–3.40 (m, 2 H), 3.98 (t, J = 6.3 Hz, 2 H), 4.57–4.61 (m, 2 H), 4.74 (s, 1 H), 6.40 (t, J = 5.5 Hz, 1 H), 6.46 (br. s., 2 H), 6.99–7.09 (m, 2 H), 7.49–7.72 (m, 3 H); Figure S16. ^13^C NMR (101 MHz, CDCl_3_) δ ppm: 14.59, 16.17, 16.19, 16.54, 18.14, 19.48, 20.97, 23.74, 25.56, 28.07, 29.43, 30.89, 32.44, 32.67, 33.70, 34.28, 37.11, 37.52, 37.66, 37.87, 38.33, 38.49, 40.09, 40.76, 42.48, 42.52, 46.74, 50.07, 50.55, 55.42, 55.61, 82.90, 109.34, 114.15, 114.82, 115.40, 122.45, 132.64, 135.57, 148.06, 150.99, 171.58, 176.32; Figure S17. HRMS-ESI: monoisotopic mass 773.52268 Da, found *m*/*z* 774.53046 [M+H]^+^ and 772.51587 [M-H]^−^; Figure S18.

*Tert*-butyl-(3-{[(3β)-3-hydroxy-28-oxolup-20(29)-ene-28-yl]amino}propyl)-carbamate (**7**) [23]

To a solution of BA (1.00 g, 2.19 mmol) and 4-DMAP (295 mg, 2.41 mmol) in DMF (12 mL), *N*-boc-1,3-diaminopropane (459 mg, 2.63 mmol), HOBt (326 mg, 2.41 mmol) and EDCI (462 mg, 2.41 mmol) were added. The mixture was stirred for 48 h at RT. The solvents were evaporated under reduced pressure and the residue was chromatographed (DCM-MeOH 100:1, *v*/*v*). Compound **7** (900 mg, 1.47 mmol) was obtained as white solid in 67% yield. R_F_ = 0.34 in DCM-MeOH 40:1 (*v*/*v*). ^1^H NMR (400 MHz, CDCl_3_) δ ppm: 0.65 0.70 (m, 1 H), 0.75 (s, 3 H), 0.81 (s, 3 H), 0.85–0.90 (m, 1 H), 0.93 (s, 3 H), 0.96 (s, 3 H), 0.97 (s, 3 H), 0.98–1.05 (m, 2 H), 1.13–1.20 (m, 1 H), 1.22–1.44 (m, 8 H), 1.45 (s, 9 H), 1.46 1.68 (m, 11 H), 1.69 (s, 3 H), 1.69–1.81 (m, 2 H), 1.88–2.00 (m, 1 H), 2.00–2.07 (m, 1 H), 2.42–2.50 (m, 1 H), 3.10–3.28 (m, 5 H), 3.30–3.39 (m, 1 H), 4.59 (s, 1 H), 4.74 (s, 1 H), 4.91 (br. s., 1 H), 6.33 (br. s., 1 H); Figure S19. ^13^C NMR (101 MHz, CDCl_3_) δ ppm: 14.64, 15.35, 16.12, 16.14, 18.28, 19.48, 20.94, 25.64, 27.41, 27.97, 28.40 (s, 3 C) 29.48, 30.54, 30.92, 33.65, 34.39, 35.28, 37.20, 37.75, 38.51, 38.72, 38.84, 40.76, 42.47, 46.66, 50.04, 50.64, 55.38, 55.76, 67.07, 78.96, 79.32, 109.27, 151.05, 156.65, 176.50; Figure S20. HRMS-ESI: calculated 612.48661 Da, found *m*/*z* 613.49394 [M+H]^+^, 635.47609 [M+Na]^+^ and 651.44943 [M+K]^+^; Figure S21.

4-{[(3β)-28-({3-[(*Tert*-butoxycarbonyl)amino]propyl}amino)-28-oxolup-20(29)-ene-3--yl]oxy}-2,2-dimethyl-4-oxobutanoic acid (**8**)

To a solution of **7** (400 mg, 0.65 mmol) and 4-DMAP (128 mg, 1.04 mmol) in THF (4 mL), 2,2-dimethylsuccinic anhydride (418 mg, 3.26 mmol) and *p*-TsOH were added. The mixture was stirred for 2 h at 130 °C in a microwave reactor. The mixture was poured into water and extracted with DCM (4 × 20 mL). Combined organic layers were washed with KHSO_4_ (3 × 5 mL) and brine. The organic layer was dried over Na_2_SO_4_ and the solvents were evaporated under reduced pressure. The residue was chromatographed (DCM-MeOH 40:1, *v/v* + 1% Et_3_N) to obtain **8** (260 mg, 0.35 mmol) as white solids in 54% yield. R_F_ = 0.18 in DCM-MeOH 40:1 (*v*/*v*) + 1% Et_3_N. ^1^H NMR (400 MHz, CDCl_3_) δ ppm: 0.74–0.77 (m, 1 H), 0.80 (s, 3 H), 0.82 (s, 6 H), 0.92 (s, 3 H), 0.96 (s, 3 H), 0.97–1.02 (m, 1 H), 1.13–1.17 (m, 1 H), 1.25–1.27 (m, 2 H), 1.28 (s, 3 H), 1.30 (s, 3 H), 1.31–1.44 (m, 7 H), 1.45 (s, 9 H), 1.46–1.68 (m, 10 H), 1.69 (s, 3 H), 1.70–1.82 (m, 2 H), 1.90–1.97 (m, 1 H), 2.03 2.07 (m, 1 H), 2.41–2.48 (m, 1 H), 2.53–2.70 (m, 2 H), 3.10–3.40 (m, 7 H), 4.46–4.51 (m, 1 H), 4.59 (s, 1 H), 4.74 (s, 1 H), 4.94 (br. s., 1 H), 6.43 (br. s., 1 H); Figure S22. ^13^C NMR (101 MHz, CDCl_3_) δ ppm: 14.61, 16.09, 16.15, 16.47, 18.14, 19.45, 20.95, 23.60, 25.02, 25.58, 25.61, 27.90, 28.39, 29.46, 30.49, 30.88, 33.61, 34.30, 35.35, 37.08, 37.70, 37.92, 38.40, 38.52, 40.44, 40.72, 40.76, 42.46, 44.71, 46.64, 49.99, 50.52, 55.47, 55.80, 79.40, 81.55, 109.37, 150.97, 156.80, 171.00, 176.72, 182.24; Figure S23. HRMS-ESI: calculated 740.53395 Da, found *m*/*z* 741.54119 [M+H]^+^ and 763.52302 [M+Na]^+^; Figure S24.

4-{[(3β)-28-[(3-Aminopropyl)amino]-28-oxolup-20(29)-ene-3-yl]oxy}-2,2-dimethyl-4-oxo-butanooic acid hydrochloride (**9**)

Compound **6** (130 mg, 0.18 mmol) was dissolved in CHCl_3_ (1.5 mL) and 2 M HCl solution in Et_2_O (3 mL) was added slowly. The mixture was stirred for 1 h at RT under argon atmosphere. Solvents were evaporated under reduced pressure and the residue was sonicated for 20 min in Et_2_O (5 mL). The product was collected by filtration and dried in vacuo. Compound **9** (102 mg, 0.16 mmol) was obtained as white solids in 91% yield. R_F_ = 0.1 in DCM-MeOH 9:1 (*v*/*v*). ^1^H NMR (400 MHz, CD_3_OD) δ ppm: 0.81–0.83 (m, 1 H), 0.86 (s, 6 H), 0.88 (s, 1 H), 0.89 (s, 3 H), 0.98 (s, 3 H), 1.02 (s, 3 H), 1.03–1.11 (m, 1 H), 1.19 (m, 1 H), 1.25 (s, 3 H), 1.26 (s, 3 H), 1.27–1.32 (m, 1 H), 1.36–1.65 (m, 15 H), 1.69 (s, 3 H), 1.70–1.76 (m, 2 H), 1.80–1.90 (m, 5 H), 2.10–2.15 (m, 1 H), 2.52–2.65 (m, 3 H), 2.90 2.96 (m, 2 H), 3.06–3.13 (m, 1 H), 3.23–3.30 (m, 2 H), 4.43–4.48 (m, 1 H), 4.59 (s, 1 H), 4.70 (s, 1 H), 7.89 (t, J = 5.9 Hz, 1 H); Figure S25. ^13^C NMR (101 MHz, CD_3_OD) δ ppm: 13.64, 15.36, 15.43, 15.66, 17.85, 18.13, 20.76, 23.25, 24.43, 24.79, 25.49, 27.06, 27.71, 29.22, 30.51, 32.59, 34.06, 35.22, 36.85, 36.86, 37.42, 37.58, 38.02, 38.03, 38.18, 39.94, 40.60, 42.13, 44.23, 49.90, 50.50, 55.46, 55.65, 81.22, 108.66, 150.77, 171.53, 179.14, 179.17; Figure S26. HRMS-ESI: calculated 640.48152 Da, found *m*/*z* 641.48955 [M+H]^+^; Figure S27.

4-{[(3β)-28-{[*N*-(4,4-Difluoro-4-bora-3a,4a-diaza-s-indacene-8-yl)-3-aminopropyl]amino}-28-oxolup-20(29)-ene-3-yl]oxy}-2,2-dimethyl-4-oxobutanoic acid (**10**)

Compound **9** (15 mg, 0.02 mmol) and **BODIPY-SMe** (6 mg, 0.03 mmol) were dissolved in the mixture of CHCl_3_ (2 mL) and THF (1 mL). To this solution, one drop of Et_3_N was added. The mixture was stirred 30 min at RT. Solvents were evaporated under reduced pressure and the residue was chromatographed (DCM-MeOH 19:1, *v/v* + 1% Et_3_N). The mixture was dissolved in AcOEt and washed with KHSO_4_ (10% solution, 3×5 mL) and brine (1 × 5 mL). Organic layer was dried over Na_2_SO_4_ and the solvents were evaporated. Compound **10** (14 mg, 0.02 mmol) as yellow-green solid in 74% yield. R_F_ = 0.73 in DCM-MeOH 9:1 (*v*/*v*). ^1^H NMR (400 MHz, CDCl_3_) δ ppm: 0.73–0.77 (m, 1 H), 0.78 (s, 3 H), 0.81 (s, 3 H), 0.82 (s, 3 H), 0.83–0.85 (m, 1 H), 0.89 (s, 3 H), 0.99 (s, 3 H), 1.00–1.10 (m, 1 H), 1.16–1.21 (m, 1 H), 1.29 (s, 3 H), 1.31 (s, 3 H), 1.34–1.67 (m, 15 H), 1.72 (s, 3 H), 1.74 1.79 (m, 2 H), 1.87–2.03 (m, 5 H), 2.49–2.71 (m, 3 H), 3.14–3.20 (m, 1 H), 3.33 (q, J = 5.4 Hz, 2 H), 3.74 (q, J = 5.5 Hz, 2 H), 4.46–4.51 (m, 1 H), 4.65 (s, 1 H), 4.77 (s, 1 H), 6.24 (t, J = 6.1 Hz, 1 H), 6.44 (br. s., 1 H), 6.51 (br. s., 1 H), 7.12 (br. s., 1 H), 7.51 (br. s., 1 H), 7.68 (br. s., 2 H), 9.71 (t, J = 5.5 Hz, 1 H); Figure S28. ^13^C NMR (101 MHz, CDCl_3_) δ ppm: 14.63, 16.11, 16.15, 16.47, 18.09, 19.43, 20.99, 23.59, 24.97, 25.62, 27.88, 29.16, 29.51, 30.86, 33.56, 34.26, 36.73, 37.06, 37.70, 37.89, 38.40, 38.49, 40.45, 40.81, 42.47, 44.40, 44.70, 46.88, 50.08, 50.47, 55.46, 55.71, 81.51, 109.83, 113.40, 114.28, 116.67, 122.36, 125.65, 131.78, 134.36, 149.06, 150.35, 171.00, 179.13, 182.28; Figure S29. HRMS-APCI: calculated 830.53291 Da, found *m*/*z* 829.52699 [M-H]^−^; Figure S30.

(3β)-28-(4-{2-[(*Tert*-butoxycarbonyl)amino]ethyl}piperazine-1-yl)-28-oxolup-20(29)-ene-3-yl acetate (**12**)

To a solution of compound **11** (1.27 g, 2.55 mmol) in DCM (20 mL), oxalyl chloride (1.2 mL) in DCM (10 mL) and 3 drops of DMF were added. After stirring for 2 h at RT, the solvents were co-evaporated with toluene (3 × 20 mL). Chloride thus obtained was dissolved in DCM (35 mL), and 1-(2-*N*-boc-aminoethyl)piperazine (876 mg, 3.82 mmol) followed by Et_3_N (0.42 mL) were added. After stirring for 16 h at RT, the mixture was diluted with DCM (20 mL) and washed with brine (3 × 30 mL). The organic layer was dried over Na_2_SO_4_ and the solvents were evaporated under reduced pressure. The residue was chromatographed (CHCl_3_-MeOH 100:1 → 50:1, *v*/*v*) to obtain product **12** (720 mg, 1.01 mmol) as white solids in 40% yield. R_F_ = 0.16 in DCM-MeOH 100:1 (*v*/*v*). ^1^H NMR (400 MHz, CDCl_3_) δ ppm: 0.77–0.80 (m, 1 H), 0.83 (s, 3 H), 0.84 (s, 3 H), 0.85 (s, 3 H), 0.94 (s, 3 H), 0.95 (s, 3 H), 0.97–1.00 (m, 1 H), 1.12–1.17 (m, 1 H), 1.28–1.42 (m, 9 H), 1.46 (s, 9 H), 1.48–1.66 (m, 7 H), 1.68 (s, 3 H), 1.70–1.74 (m, 1 H), 1.81–1.87 (m, 1 H), 1.93–1.98 (m, 1 H), 2.04 (s, 3 H), 2.07–2.11 (m, 1 H), 2.42 (br. s., 4 H), 2.49 (br. s., 2 H), 2.83–2.90 (m, 1 H), 2.94–3.01 (m, 1 H), 3.25 (br. s., 2 H), 3.61 (br. s., 4 H), 4.44–4.49 (m, 1 H), 4.58 (s, 1 H), 4.72 (s, 1 H), 4.98 (br. s., 1 H); Figure S31. ^13^C NMR (101 MHz, CDCl_3_) δ ppm: 14.62, 16.11, 16.24, 16.46, 18.18, 19.63, 21.15, 21.30, 23.70, 25.61, 27.93, 28.41, 29.79, 31.30, 32.46, 32.49, 34.31, 35.91, 36.86, 36.95, 37.14, 37.80, 38.41, 40.68, 41.85, 45.65, 50.76, 52.65, 53.12, 54.52, 55.52, 57.15, 79.30, 80.97, 109.16, 151.30, 155.90, 170.99, 173.46; Figure S32. HRMS-ESI: calculated 709.53937 Da, found *m*/*z* 710.54562 [M+H]^+^ and 732.52546 [M+Na]^+^; Figure S33.

*Tert*-butyl-(2-{4-[(3β)-3-hydroxy-28-oxolup-20(29)-ene-28-yl]piperazine-1-yl}ethyl)-carbamate (**13**)

To compound **12** (700 mg, 0.99 mmol) in MeOH (18 mL) and THF (9 mL), 4 M NaOH solution (9 mL) was added. The mixture was stirred for 2 h at RT. The mixture was neutralized by 1 M HCl solution and extracted with DCM (4 × 40 mL). Combined organic layers were washed with saturated brine (2 × 50 mL) and dried over Na_2_SO_4_. Solvents were evaporated under reduced pressure and the residue was chromatographed (DCM-MeOH 40:1, *v*/*v*) to obtain **13** (310 mg, 0.46 mmol) as white solids in 47% yield. R_F_ = 0.33 in DCM-MeOH 20:1 (*v*/*v*). ^1^H NMR (400 MHz, CDCl_3_) δ ppm: 0.66–0.69 (m, 1 H), 0.75 (s, 3 H), 0.82 (s, 3 H), 0.86–0.90 (m, 1 H), 0.93 (s, 3 H), 0.96 (br. s, 6 H), 1.12–1.17 (m, 1 H), 1.23–1.29 (m, 2 H), 1.29–1.42 (m, 8 H), 1.45 (s, 9 H), 1.48–1.66 (m, 7 H), 1.68 (s, 3 H), 1.69–1.73 (m, 1 H), 1.81–1.87 (m, 1 H), 1.93–1.98 (m, 1 H), 2.06–2.11 (m, 1 H), 2.42 (br. s., 4 H), 2.48 (br. s., 2 H), 2.84–2.90 (m, 1 H), 2.94–3.00 (m, 1 H), 3.15–3.20 (m, 1 H), 3.25 (br. s, 2 H), 3.61 (br. s., 4 H), 4.57 (s, 1 H), 4.72 (s, 1 H), 4.99 (br. s., 1 H); Figure S34. ^13^C NMR (101 MHz, CDCl_3_) δ ppm: 14.67, 15.34, 16.11, 16.18, 18.30, 19.66, 21.14, 25.66, 27.43, 27.98, 28.41, 29.80, 31.32, 32.48, 32.50, 34.39, 35.91, 36.88, 36.97, 37.23, 38.74, 38.85, 40.67, 41.87, 45.62, 50.86, 52.67, 53.11, 54.53, 55.47, 57.15, 78.97, 79.28, 109.10, 151.33, 155.91, 173.45; Figure S35. HRMS-ESI: calculated 667.52881 Da, found *m*/*z* 668.53693 [M+H]^+^; Figure S36.

(3β)-28-[4-(2-Aminoethyl)piperazine-1-yl]-3-hydroxylup-20(29)-ene-28-one hydrochloride (**14**)

To compound **13** (200 mg, 0.30 mmol) in CHCl_3_ (2.8 mL), 2 M HCl solution in Et_2_O (5.1 mL) was slowly added. The mixture was stirred for 16 h at RT under argon. Solvents were removed under reduced pressure and the crude was sonicated for 20min in Et_2_O (5 mL), collected by filtration and dried. Compound **14** (142 mg, 0.25 mmol) was isolated as white solid in 84% yield. ^1^H NMR (400 MHz, CD_3_OD) δ ppm: 0.69–0.73 (m, 1 H), 0.75 (s, 3 H), 0.86 (s, 3 H), 0.88–0.93 (m, 1 H), 0.95 (s, 3 H), 0.96 (s, 3 H), 1.01 (s, 3 H), 1.02–1.07 (m, 1 H), 1.22–1.67 (m, 18 H), 1.70 (s, 3 H), 1.73–1.77 (m, 1 H), 1.82–1.88 (m, 1 H), 1.97–2.02 (m, 1 H), 2.11–2.15 (m, 1 H), 2.79–2.86 (m, 1 H), 2.89–2.95 (m, 1 H), 3.11–3.15 (m, 1 H), 3.35–3.81 (m, 11 H), 4.59 (s, 1 H), 4.70 (s, 1 H); Figure S37. ^13^C NMR (101 MHz, CD_3_OD) δ ppm: 13.65, 14.68, 15.27, 15.38, 18.04, 18.29, 20.86, 25.52, 26.63, 26.67, 27.18, 29.66, 30.92, 31.88, 33.62, 33.63, 34.19, 36.94, 36.96, 38.53, 38.68, 40.54, 41.64, 45.78, 50.78, 52.19, 52.37, 53.15, 54.58, 55.52, 78.24, 108.66, 150.85, 174.45; Figure S38. HRMS-ESI: calculated 567.47638 Da, found *m*/*z* 568.4832 [M+H]^+^; Figure S39.

(3β)-28-{4-[*N*-(4,4-Difluoro-4-bora-3a,4a-diaza-*s*-indacene-8-yl)-2-aminoethyl]piperazine-1-yl}-3-hydroxylup-20(29)-ene-28-one (**15**)

To compound **14** (45 mg, 0.08 mmol) and **BODIPY-SMe** (21 mg, 0.09 mmol) in the mixture of DCM (5 mL) and THF (2.5 mL), was added one drop of Et_3_N and the mixture was stirred 30 min at RT. Solvents were evaporated under reduced pressure and the residue was chromatographed (DCM-MeOH 100:1, *v*/*v*) to obtain **15** (42 mg, 0.06 mmol) as yellow solids in 70% yield. R_F_ = 0.19 in DCM-MeOH 100:1 (*v*/*v*). ^1^H NMR (400 MHz, CDCl_3_) δ ppm: 0.67–0.71 (m, 1 H), 0.76 (s, 3 H), 0.83 (s, 3 H), 0.87–0.91 (m, 1 H), 0.95 (s, 3 H), 0.97 (s, 6 H), 1.16–1.21 (m, 1 H), 1.21–1.32 (m, 3 H), 1.38–1.66 (m, 15 H), 1.69 (s, 3 H), 1.72–1.76 (m, 1 H), 1.82–1.89 (m, 1 H), 1.93–1.98 (m, 1 H), 2.06–2.12 (m, 1 H), 2.55 (br. s., 4 H), 2.82–2.90 (m, 3 H), 2.95–3.01 (m, 1 H), 3.17–3.21 (m, 1 H), 3.70 (br. s., 4 H), 3.75 (br. s., 2 H), 4.60 (s, 1 H), 4.74 (s, 1 H), 6.41 (br. s., 1 H), 6.53 (br. s., 1 H), 6.91 (br. s., 1 H), 7.13 (br. s., 1 H), 7.51 (br. s., 1 H), 7.72 (br. s., 1 H), 7.92 (br. s., 1 H); Figure S40. ^13^C NMR (101 MHz, CDCl_3_) δ ppm: 14.68, 15.34, 16.18, 18.32, 19.64, 21.14, 25.45, 25.64, 27.43, 27.97, 29.86, 31.31, 32.52, 34.42, 35.93, 36.92, 37.24, 38.74, 38.86, 40.69, 41.80, 41.90, 45.62, 50.84, 52.53, 52.65, 54.26, 54.60, 55.46, 78.98, 109.29, 113.76, 114.93, 122.67, 123.28, 125.04, 132.47, 135.78, 147.77, 151.12, 173.64; Figure S41. HRMS-ESI: calculated 757.52776 Da, found *m*/*z* 780.5171 [M+Na]^+^ and 796.4904 [M+K]^+^; Figure S42.

4-{[(3β)-28-(4-{2-[(*Tert*-butoxycarbonyl)amino]ethyl}piperazine-1-yl)-28-oxolup-20(29)-ene-3-yl]oxy}-2,2-dimethyl-4-oxobutanoic acid (**16**)

To a solution of **13** (500 mg, 0.75 mmol) and 4-DMAP (146 mg, 1.20 mmol) in THF (5 mL), 2,2-dimethylsuccinic anhydride (480 mg, 3.74 mmol) and a catalytic amount of *p*-TsOH were added. The reaction was stirred for 2 h at 130 °C in a microwave reactor. The mixture was diluted with H_2_O and extracted with DCM (4 × 20 mL). Combined organic layers were dried over Na_2_SO_4_ and the solvents were removed under reduced pressure. The crude was chromatographed twice (i. toluene-AcOEt 1:1 → AcOEt; ii. toluene-AcOEt 1:1 + 1% Et_3_N → AcOEt → DCM-MeOH 9:1, *v*/*v*). Compound **16** (328 mg, 0.41 mmol) was obtained as white solids in 53% yield. R_F_ = 0.43 in DCM-MeOH 9:1 (*v*/*v*). ^1^H NMR (400 MHz, CDCl_3_) δ ppm: 0.74–0.77 (m, 1 H), 0.81 (s, 6 H), 0.83 (s, 3 H), 0.93 (s, 3 H), 0.95 (s, 3 H), 1.12–1.16 (m, 1 H), 1.27 (br. s., 6 H), 1.32–1.41 (m, 8 H), 1.44 (s, 9 H), 1.47–1.65 (m, 7 H), 1.68 (s, 3 H), 1.70–1.74 (m, 1 H), 1.80–1.86 (m, 1 H), 1.90–1.95 (m, 1 H), 2.05–2.09 (m, 1 H), 2.49–2.69 (m, 6 H), 2.82–2.89 (m, 1 H), 2.93–3.00 (m, 1 H), 3.29 (br. s., 2 H), 3.61 (br. s., 4 H), 3.71 (br. s., 2 H), 4.45–4.50 (m, 1 H), 4.58 (s, 1 H), 4.72 (s, 1 H), 5.15 (br. s., 1 H); Figure S43. ^13^C NMR (101 MHz, CDCl_3_) δ ppm: 14.67, 16.06, 16.21, 16.57, 18.23, 19.64, 21.14, 23.66, 23.68, 25.57, 25.58, 25.92, 25.94, 27.91, 28.40, 29.82, 31.30, 32.36, 32.39, 34.26, 35.96, 36.69, 36.82, 37.12, 37.76, 38.39, 38.41, 40.63, 41.87, 45.60, 50.72, 52.60, 52.94, 54.49, 55.53, 57.19, 57.20, 109.24, 128.19, 129.00, 151.20, 155.99, 173.43; Figure S44. HRMS-ESI: calculated 795.57615 Da, found *m*/*z* 796.58292 [M+H]^+^; Figure S45.

4-{[(3β)-28-[4-(2-Aminoethyl)piperazine-1-yl]-28-oxolup-20(29)-ene-3-yl]oxy}-2,2-dimethyl-4-oxobutanoic acid hydrochloride (**17**)

To compound **16** (150 mg, 0.19 mmol) in CHCl_3_ (2 mL), 2 M HCl solution in Et_2_O (3.2 mL) was added. The mixture was stirred for 2 h at RT under argon atmosphere. Solvents were evaporated and the crude was sonicated for 20 min in Et_2_O (5 mL). The precipitate was collected and dried in vacuo. Compound **17** (102 mg, 0.16 mmol) was obtained as white solids in 91% yield. ^1^H NMR (400 MHz, CD_3_OD) δ ppm: 0.82–0.85 (m, 1 H), 0.87 (br. s, 6 H), 0.91 (s, 3 H), 0.98 (s, 3 H), 0.99–1.02 (m, 1 H), 1.04 (s, 3 H), 1.05–1.10 (m, 1 H), 1.27 (s, 3 H), 1.28 (s, 3 H), 1.29–1.69 (m, 8 H), 1.71 (s, 3 H), 1.73–1.78 (m, 2 H), 1.83–1.89 (m, 1 H), 1.98–2.04 (m, 1 H), 2.13–2.18 (m, 1 H), 2.54–2.67 (m, 2 H), 2.81–2.87 (m, 1 H), 2.91–2.97 (m, 1 H), 3.05–3.77 (m, 11 H), 4.45–4.49 (m, 1 H), 4.61 (s, 1 H), 4.72 (s, 1 H); Figure S46. ^13^C NMR (101 MHz, CD_3_OD) δ ppm: 13.72, 15.25, 15.40, 15.67, 17.87, 18.29, 20.87, 20.90, 23.27, 24.44, 24.79, 25.43, 25.45, 27.09, 29.66, 30.91, 31.85, 33.65, 34.06, 36.89, 36.92, 37.44, 38.21, 39.94, 40.54, 41.67, 44.24, 45.78, 50.65, 52.19, 52.32, 53.16, 54.56, 55.52, 81.25, 108.69, 150.83, 171.53, 174.40, 179.15; Figure S47. HRMS-ESI: calculated 695.52372 Da, found *m*/*z* 696.53153 [M+H]^+^; Figure S48.

4-{[(3β)-28-{4-[*N*-(4,4-Difluoro-4-bora-3a,4a-diaza-*s*-indacene-8-yl)-2-aminoethyl]piperazine-1-yl}-28-oxolup-20(29)-ene-3-yl]oxy}-2,2-dimethyl-4-oxobutanoic acid (**18**)

To compound **17** (50 mg, 0.07 mmol) and **BODIPY-SMe** (19 mg, 0.08 mmol) in the mixture of CHCl_3_ (5 mL) and THF (3 mL), a drop of Et_3_N was added. The mixture was stirred 30 min at RT. Solvents were evaporated and the residuum was chromatographed (DCM-MeOH 20:1, *v/v* + 0.5% Et_3_N). Compound **18** (39 mg, 0.04 mmol) was obtained as yellowish solid in 62% yield. R_F_ = 0.22 in DCM-MeOH 20:1 (*v*/*v*) + 0.5% Et_3_N. ^1^H NMR (400 MHz, CDCl_3_) δ ppm: 0.75–0.78 (m, 1 H), 0.81 (s, 3 H), 0.83 (s, 3 H), 0.84 (s, 3 H), 0.88 0.92 (m, 1 H), 0.94 (s, 3 H), 0.96 (s, 3 H), 1.15–1.19 (m, 1 H), 1.28 (s, 3 H), 1.30 (s, 3 H), 1.35 1.42 (m, 7 H), 1.45–1.51 (m, 2 H), 1.51–1.64 (m, 6 H), 1.69 (s, 3 H), 1.72–1.76 (m, 1 H), 1.83 1.89 (m, 1 H), 1.93–1.98 (m, 1 H), 2.06–2.12 (m, 1 H), 2.51–2.70 (m, 6 H), 2.82–2.89 (m, 3 H), 2.94–3.00 (m, 1 H), 3.68 (br. s., 4 H), 3.75 (br. s., 2 H), 4.46–4.51 (m, 1 H), 4.59 (s, 1 H), 4.73 (s, 1 H), 6.39 (br. s., 1 H), 6.52 (br. s., 1 H), 6.91 (br. s., 1 H), 7.13 (br. s., 1 H), 7.50 (br. s., 1 H), 7.71 (br. s., 1 H), 7.96 (br. s., 1 H); Figure S49. ^13^C NMR (101 MHz, CDCl_3_) δ ppm: 14.67, 16.13, 16.21, 16.50, 18.18, 19.60, 21.17, 23.65, 25.05, 25.60, 27.92, 29.68, 29.84, 31.29, 32.49, 34.32, 35.94, 36.88, 37.12, 37.74, 38.42, 40.48, 40.68, 41.88, 41.96, 44.75, 45.65, 50.71, 52.57, 52.61, 54.28, 54.57, 55.55, 81.47, 109.33, 113.68, 114.87, 122.61, 123.38, 125.04, 132.41, 135.68, 147.79, 151.11, 171.11, 173.64, 182.29; Figure S50. HRMS-ESI: calculated 885.57511 Da, found *m*/*z* 884.56855 [M-H]^−^; Figure S51.

### 2.2. Biochemistry

#### 2.2.1. Cell Lines

All cells (if not indicated otherwise) were purchased from the American Tissue Culture Collection (ATCC; Manassas, VA, USA). The highly chemosensitive CCRF-CEM line is derived from T lymphoblastic leukaemia, K562 represent cells of chronic myelogenous leukaemia. Colorectal adenocarcinoma HCT116 cell line and its p53 gene knockout counterpart (HCT116p53−/−, Horizon Discovery Ltd., Cambridge, UK) were used as models to assess the impact of p53 deficiency on cell line sensitivity. A549 cells are derived from lung adenocarcinoma and U2OS from human osteosarcoma. CEM-DNR and K562-Tax are well-characterized daunorubicin and paclitaxel-resistant sublines of CCRF-CEM and K562. The CEM-DNR resistant cells overexpress the P-glycoprotein and LRP protein, the K562-Tax overexpress P-glycoprotein but is losing the expression of LRP, which is present at parental K562 cell line. P-glycoprotein belongs to the ABC transporters’ family and is involved in the primary and acquired multidrug resistance phenomenon by the efflux of toxic compounds, LRP protein is involved in the lysosomal degradation. MRC-5 and BJ cell lines were used as a non-tumour control and represent human fibroblasts. The cells were maintained in Nunc/Corning 80 cm^2^ plastic tissue culture flasks and cultured in cell culture medium according to ATCC or Horizon recommendations (DMEM/RPMI 1640 with 5 g/L-glucose, 2 mM glutamine, 100 U/mL penicillin, 100 mg/mL streptomycin, 10% fetal calf serum, and NaHCO_3_).

#### 2.2.2. MTS Assay

To perform the cytotoxicity MTS assay, cell suspensions were prepared and diluted according to the cell type and the expected target cell density (25,000–35,000 cells/mL) based on cell growth characteristics. Cells were added by an automatic pipettor (30 μL) into 384 well microtiter plates. All tested compounds were dissolved in 100% DMSO and four-fold dilutions of the intended test concentration were added in 0.15 μL aliquots at time zero to the microtiter plate wells by the echo-acoustic liquid handler Echo550 (Labcyte, San Jose, CA, USA). The experiments were performed in technical duplicates and at least three biological replicates. The cells were incubated with the tested compounds for 72 h at 37 °C, in a 5% CO_2_ atmosphere at 99% humidity. At the end of the incubation period, the cells were assayed by using the MTS test. Aliquots (5 µL) of the MTS stock solution were pipetted into each well and incubated for an additional 1–4 h. After this incubation period, the optical density (OD) was measured at 490 nm with an Envision microplate reader (Perkin Elmer, Waltham, Massachusetts, USA). Tumour cell survival (TCS) was calculated using the following equation: TCS = (OD_drug-exposed well_/mean OD_control wells_) × 100%. The IC_50_ value, the drug concentration that is lethal to 50% of the tumour cells, was calculated from the appropriate dose-response curves in Dotmatics software (The Old Monastery, Windhill, Bishop´s Stortford, Herts, UK).

#### 2.2.3. Cell Cycle and Apoptosis Analysis

CCRF-CEM cells were seeded in 6-well plates at a density of 1 × 106/well. After 24 h, compounds at concentrations corresponding to 1× or 5 × IC_50_ were added to the wells and incubated for 24 h. Cells were then harvested, washed with cold 1 × PBS and fixed in ice-cold 70% ethanol. Fixed cells were incubated overnight at −20 °C, washed in hypotonic citrate buffer, treated with RNase (50 μg mL^−1^) and incubated with propidium iodide for 15 min. DNA content was analysed using Becton Dickinson flow cytometer and cell cycle data were analysed in the program ModFitLT (Verity, Carrollton, TX, USA). Apoptosis was measured in a logarithmic model expressing the percentage of the particles with propidium content lower than cells in G0/G1 phase (<G1) of the cell cycle. The mitotic marker pH3Ser10 antibody (Sigma) and secondary anti-mouse-FITC antibody (Sigma) were used for labelling and subsequent flow cytometry analysis of ethanol-fixed CCRF-CEM cells.

#### 2.2.4. BrDU Incorporation Analysis

Cells were cultivated as in the method above and pulse-labelled with 10 μM 5-bromo-2-deoxyuridine (BrDU) for 30 min before collection to the test tubes. The cells were washed with cold 1 × PBS and fixed in ice-cold 70% ethanol. Before analysis, they were washed with 1 × PBS and incubated in 2M HCl for 30 min at room temperature. Following neutralization with 0.1M Na_2_B_4_O_7_ (borax), the cells were washed with 0.5% Tween-20 and 1% BSA in 1 × PBS. The cell pellets were stained using a primary anti-BrdU antibody (Exbio, Vestec, Czech Republic) for 30 min at room temperature and a secondary anti-mouse-FITC antibody (Sigma). The samples were then incubated with propidium iodide (0.1 mg mL^−1^), treated with RNase A (0.5 mg mL^−1^) for 1 h at room temperature in the dark and analysed as above.

#### 2.2.5. BrU Incorporation Analysis

Cells were cultured, treated as above, pulse-labelled with 1 mM 5-bromouridine (BrU) for 30 min and fixed in 1% buffered paraformaldehyde with 0.05% NP-40 at room temperature for 15 min. Following overnight incubation at 4 °C, they were washed with 1% glycine in 1 × PBS, washed with 1 × PBS again and stained with primary anti-BrdU antibody cross-reacting to BrU (Exbio) for 30 min and secondary anti-mouse-FITC antibody (Sigma). The analysis was performed similarly to the BrDU analysis.

#### 2.2.6. Fluorescent Microscopy

U2OS cell line (ATCC, USA) was transduced with premade lentiviral particles (Vectalis-TaKaRa, Japan) with sequences that express fluorescent protein tag mCherry targeted to specific subcellular locations. All cell lines were prepared according to the vendor’s instructions. The U2OS-Nuc cell line was prepared by using rLV.EF1.mCherry-Nuc-9 (cat. n. 0023VCT), containing a NLS sequence that imports protein into the nucleus. The U2OS-ER cell line was transduced by rLV.EF1.mCherry-ER-9 (cat. n. 0025VCT), which contains a calreticulin signal sequence and a KDEL sequence that associates protein with the endoplasmic reticulum. The U2OS-GA cell line was transduced by rLV.EF1.mCherry-Golgi-9 (cat. n. 0022VCT), containing a human GT precursor, a protein localized in Golgi Apparatus. The U2OS-Mito cell line was prepared by using rLV.EF1.mCherry-Mito-9 (cat. n. 0024VCT), containing a mitochondrial targeting sequence.

U2OS cells with fluorescent fusion proteins (density 1.0 × 103 per well) were seeded into 384 CellCarrier plates (Perkin Elmer, Waltham, MA, USA) and pre-incubated for 24 h at 37 °C and 5% CO_2_. The attached cells were treated with tested compounds in concentration 10 µM for 1 h and subsequently rinsed with fresh media. The live-cell imaging was performed by Cell Voyager CV7000 (Yokogawa, Tokyo, Japan) spinning disc confocal microscopy system at 37 °C in a 5% CO_2_ atmosphere. Live cells were monitored by a 60 × water immersion objective. The fluorescent signal was excited by lasers (405 nm and 561 nm) and the emission was filtered by bandpass filters (BP 445/45 and BP 595/20). All images were post-processed, and Pearson’s and Mander’s coefficients were calculated using the JACoP plugin in Image-J software.

#### 2.2.7. VSV-G Pseudotyped HIV-1 Particles Production

HIV-1 particles were obtained from HEK 293 cells, cotransfected by a combination of three vectors: packaging psPAX2 vector encoding HIV Gag, Pol, Tat and Rev, reporter/transfer pWPXLd-GFP vector encoding LTR, RRE and GFP as a reporter, and envelope pHEF-VSV-G vector, encoding vesicular stomatitis virus Env, VSV-G. The psPAX2 vector [32] was kindly provided by Dr. Luban, the pWPXLd-GFP and pHEF-VSV-G vectors were purchased from Addgene (Watertown, MA, USA).

HEK-293 cells were grown in Dulbecco’s Modified Eagle Medium (DMEM, Sigma) supplemented with 10% fetal bovine serum (Sigma) and 1% L-glutamine (Sigma) at 37 °C under 5% CO_2_. A day before transfection, cells were plated at 3 × 105 cells per well. The following day, cells were transfected with the appropriate vectors using polyethylenimine (PEI, 1 mg/mL) at a 2:1 PEI:DNA ratio. Four hours post-transfection, the culture medium was replaced with fresh DMEM, containing various concentrations of tested compounds, solubilized in DMSO. At 48 h post-transfection, the culture media containing released virions were harvested, filtered through 0.45-µm pores membrane and used for immunochemical quantification and characterization by ELISA and Western blot using rabbit anti-HIV-1 CA antibody.

#### 2.2.8. Single-Round Infectivity Assay

The infectivity was determined similarly as described earlier [33,34,35]. Briefly, 48 h post-transfection, the culture media from HEK 293 cells transfected with psPAX2, pWPXLd-GFP and pHEF-VSV-G vectors at a ratio 1:1:1 in the presence of tested compounds were collected and filtered through a 0.45-µm filter. HIV-1 CA content was determined by ELISA [33]. The freshly seeded HEK 293 cells were infected with ELISA-normalized amounts of VSV-G pseudotyped HIV-1 particles and incubated for 48 h. The cells were fixed with 2% paraformaldehyde and transferred to a FACS tube. Quantification of GFP-positive cells was performed using a BD FACS Aria III flow cytometer (BD Life Sciences, San Jose, CA, USA).

#### 2.2.9. Western Blot

At 48 h post-transfection, 100 µL aliquots of virus-containing culture media were combined with 20 µL of PLB (6×) and the samples were analysed by Western blot using rabbit anti-HIV-1 CA (in house production). Proteins were resolved by reducing SDS-PAGE (12%) and blotted onto a nitrocellulose membrane. The antigen-antibody complexes were detected by Clarity™ Western ECL Substrate (Biorad, Hercules, CA, USA) and visualized using the FUSION 7S system (Vilber Lourmat, Marne-la-Vallée, France).

## 3. Results and Discussion

### 3.1. Chemistry

**Figure 2:**
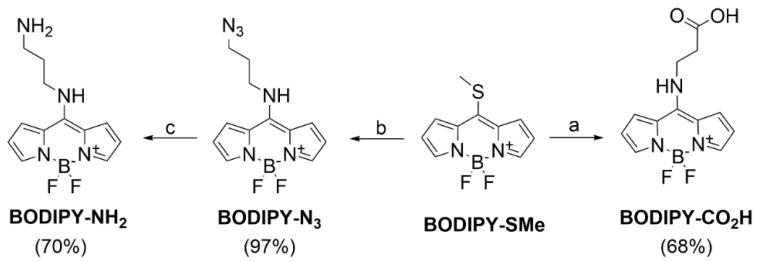
Synthesis of functionalized BODIPY dyes. Reagents and conditions: (**a**) β-Ala, DMSO-H_2_O, 30 °C, 12 h; (**b**) 3-azidopropylamine, DCM, 30 min, RT; (**c**) H_2_, Pd/C, AcOEt, 2h, RT.

The synthesis of fluorescent labels was based on 8-thiomethyl BODIPY (**BODIPY-SMe**; Figure 2), which was prepared in our laboratory, according to the procedure previously described in the literature [36]. The thiomethyl group is reactive towards amines. After this reaction, secondary amines are formed with significant fluorescence characterized by emission in the blue region of the spectrum. For the preparation of betulinic acid conjugates, a carboxy-terminated derivative (**BODIPY-CO_2_H**, Figure 2) was prepared by reaction of **BODIPY-SMe** and β-alanine [37] and an amino-terminated derivative by reaction with 3-azidopropan-1-amine [38] and reduction of azide (**BODIPY-N_3_**, Figure 2) to amine (**BODIPY-NH_2_**, Figure 2) by catalytic hydrogenation.

**Figure 3:**
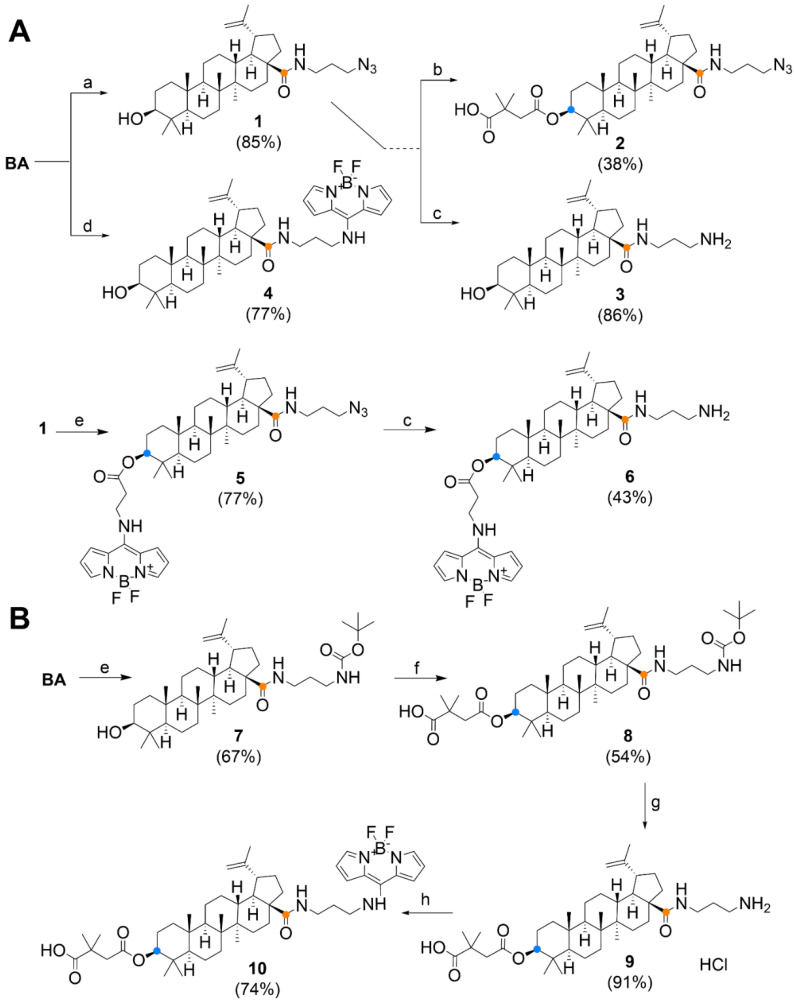
Synthesis of “aminopropyl” derivatives by azide reduction (panel **A**) and Boc chemistry (panel **B**). Reagents and conditions: (a) 3-azidopropylamine, 4-DMAP, HOBt, EDC, DMF, 36 h, RT; (b) 2,2-dimethylsuccinic anhydride, 4-DMAP, *p*-TsOH, THF, 2 h, MW-130 °C; (c) PPh_3_, THF/H_2_O, 23 h; (d) BODIPY-NH_2_, 4-DMAP, DCC, DCM, 12 h, RT; (e) BODIPY-CO_2_H, DCC, 4-DMAP, DCM, 12 h, RT; (f) *N*-Boc-1,3-diaminopropane, EDC, HOBt, 4-DMAP, DMF, 48 h, RT; (g) 2M HCl/Et_2_O, 12 h, RT (under argon); (h) BODIPY-SMe, CHCl_3_-THF, Et_3_N, 30 min, RT.

Betulinoyl azidopropylamide (*N*-(3-azidopropyl)-3β-hydroxylup-20(29)-en-28-amide) **1** was prepared by reacting BA with 3-azidopropan-1-amine using carbodiimide chemistry (Figure 3A). The reaction was catalysed by EDCI (1-ethyl-3-(3-dimethylaminopropyl)carbodiimide) in the presence of 4-DMAP (4-dimethylaminopyridine) and HOBt (1-hydroxybenzotriazole). The bevirimat derivative **2** was prepared from compound **1** by reaction with 2,2-dimethylsuccinic anhydride according to a protocol reported in the literature [39]. By Staudinger reduction [40] catalysed by triphenylphosphine in aqueous THF, the azido group of compound **1** was reduced to amino derivative **3**. In an effort to reduce derivative **2** by the same method, a non-separable mixture of products was obtained. By the reaction of BA with **BODIPY-NH_2_** catalysed by DCC (*N*,*N′*-dicyclohexylcarbodiimide) in the presence of 4-DMAP, derivative **4** was obtained. This reaction proceeded without difficulty in good yield (Figure 3A). Azide 1 was further conjugated at the C-3 position with **BODIPY-CO_2_H** by Steglich esterification [41] to produce derivative **5**. The azide group of derivative **5** was reduced by Staudinger reduction to amine **6**. When attempting to modify compound **4** with dimethylsuccinic anhydride under the conditions used to prepare derivative **2**, degradation of the fluorescent label occurred, probably due to too high a temperature. Therefore, another synthetic procedure using a *tert*-butoxycarbonyl protecting group (Boc) on the terminal amino group was chosen for the synthesis of other “aminopropyl” derivatives (Figure 3B). The *N*-Boc-1,3-diaminopropane linker was conjugated to BA to give compound **7**, which could already be used to prepare the bevirimat derivative **8**. The protecting group was removed in an acidic environment to give amine **9**. From compound **9**, a fluorescently labelled derivative of bevirimat was prepared by the reaction with **BODIPY-SMe**.

**Figure 4:**
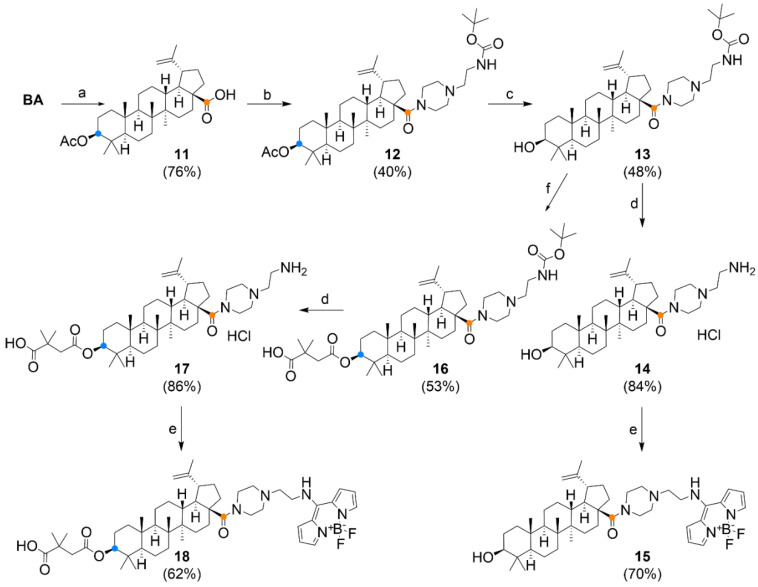
Synthesis of “piperazinyl” derivatives. Reagents and conditions: (a) Ac_2_O, pyridine, 12 h, RT; (b) i. (COCl)_2_, DCM, DMF, 2 h, RT, ii. 1-(2-*N*-boc-aminoethyl)piperazine, Et_3_N, DCM, 12 h, RT; (c) 4M NaOH, THF-MeOH, 3 h, RT; (d) 2M HCl/Et_2_O, 12 h, RT (under argon); (e) BODIPY-SMe, CHCl_3_-THF, Et_3_N, 30 min, RT; (f) 2,2-dimethyl succinic anhydride, 4-DMAP, *p*-TsOH, THF, 2 h, MW-130 °C.

Analogous to the synthetic procedure shown in Figure 3B, a series of substances with a piperazine linker at position C-28 was prepared (Figure 4). The introduction of the piperazine motif was chosen on the basis of promising results for the so-called privileged structures published previously [21]. The exception was that the C-3 hydroxyl was first acetylated to produce compound **11**, and in the next step, the carboxyl group was activated to reactive chloride. After condensation with 1-(2-*N*-Boc-aminoethyl) piperazine, tertiary amide **12** was obtained. Deacetylation of **12** occurred in a relatively low yield; however, part of the starting material was recovered during the separation of the reaction mixture.

Experimental details of the preparation of substances are described in Section 2.1. and the NMR, HRMS spectra (Figures S1–S51) and photochemical properties (Figure S52 and Table S1) of the substances are shown in the Supplementary Material.

### 3.2. Cytotoxicity on a Panel of Cell Lines

The in vitro cytotoxicity of derivatives was assessed using MTS assay on the normal human foreskin and lung fibroblasts BJ and MRC-5 and cancer cell lines of a different histogenetic type (Table 1). Under the experimental conditions, BA and BT showed a weak or medium cytotoxic effect directed against cancer cell lines. Structures **5**, **15** and **16** did not induce any cytotoxic effect in the entire cell line panel at the maximal tested concentration. Derivatives **4**, **9**, **12**, **17** and **18** were inactive against the entire cell line panel except for the CCRF-CEM lymphoblastic leukaemia cell line. IC_50_ values obtained for these compounds in the sensitive cell line CCRF-CEM were between 5.76 and 23.65 µM. Derivatives **3**, **6**, **13** and **14** exerted high cytotoxicity against the entire cell line panel, including normal fibroblasts. The most potent compounds in the study were structures **3** and **14** with IC_50_ values 0.21 and 0.29 µM in CCRF-CEM. Derivatives **2**, **8** and **10** displayed medium cytotoxicity across the cell line panel. Derivatives **1** and **7** showed activity only against selected cell lines in the panel. Betulinic acid intermediate **11** was not tested. The MTS assay did not reveal any effect directed specifically against cancer cell lines, IC_50_ values calculated for normal fibroblast and cancer cell lines were highly comparable. Resistant sublines CEM-DNR and K562-Tax displayed for some compounds different sensitivity compared to their parental cell lines. As expected, a lower sensitivity was observed in the CEM-DNR resistant subline. The biggest difference in favour of CEM-DNR was observed for derivatives **6** and **3**. **BA** and **13** showed an opposite profile in CEM-DNR and **1**, **2**, **3**, **8** and **10** in the K562-Tax resistant subline, proposing better activity in resistant cell lines. Based on this data, we can speculate that there is a different mechanism in the elimination of cytotoxic derivatives. Several tested compounds are probably substrates of the P-glycoprotein as **4**, **6**, **13**, **16**, **17** and **18**. However, not all data are in conclusion with P-glycoprotein transport, and we think that several tested derivatives could be substrates for LRP protein. Higher LRP expression in CEM-DNR and lower in K562-Tax correlates with cytotoxicity of the derivatives **1**, **2**, **3**, **8**, **10**. Derivative **13** is not active in the highly chemosensitive CCRF-CEM cell line, but comparable activity was observed in all tested cell lines, including non-tumour lines.

### 3.3. Cell Cycle, Apoptosis and DNA/RNA Synthesis

To reveal cytostatic effects, we examined proliferation markers and cell cycle profile of the sensitive CCRF-CEM cell line following a 24 h incubation with the derivatives (Table 2).

Exposure to 1 × IC_50_ and 5 × IC_50_ concentrations of derivatives did not induce DNA fragmentation with the exception of high doses of **2** and **8**. Treatment with 1 × IC_50_ concentrations did not modulate cell cycle profile while 5 × IC_50_ concentration led in all samples to a more pronounced effect. The treatment with 5 × IC_50_ derivatives **2**, **12** and **14** increased the percentage of cells in the S-phase by about 50% compared to untreated control. Nevertheless, there was not any other prominent effect on the cell cycle profile or cell cycle arrest. To assess the impact of structures on CCRF-CEM proliferation potential, we monitored mitotic marker pH3Ser10 and proliferation marker BrDU after 24 h incubation with the compounds. Analysis of mitotic marker showed a low rate of cell division in cells treated with 5 × IC_50_ concentration of derivatives **2**, **3**, **6**, **12** and **14**. Derivatives **3**, **6**, **8**, **10**, and **14** reduced the fraction of proliferating BrDU positive CCRF-CEM cells. In contrast, structures **2**, **12** and **18** increased the percentage of cells incorporating BrDU into the DNA during pulse labelling. The complementary BrU based method of monitoring newly synthesized RNA in cells pre-incubated for 24 with the selected derivatives revealed stalled RNA synthesis induced by 5 × IC_50_ concentration of derivatives **2**, **3**, **8**, **10**, **14** and **17**. Compound **18** at a high concentration increased the percentage of BrU positive cells. Such an increase indicates the high transcription activity as a mark of replication stress leading to DNA damage and cell death [42]. Although there was observed a slight modulation of cell cycle profile induced by compound derivatives **2**, **12** and **14**, the overall cell cycle data indicates that there is no general cytostatic effect of tested betulinic acid derivatives.

### 3.4. Live Cells Imaging

**Figure 5:**
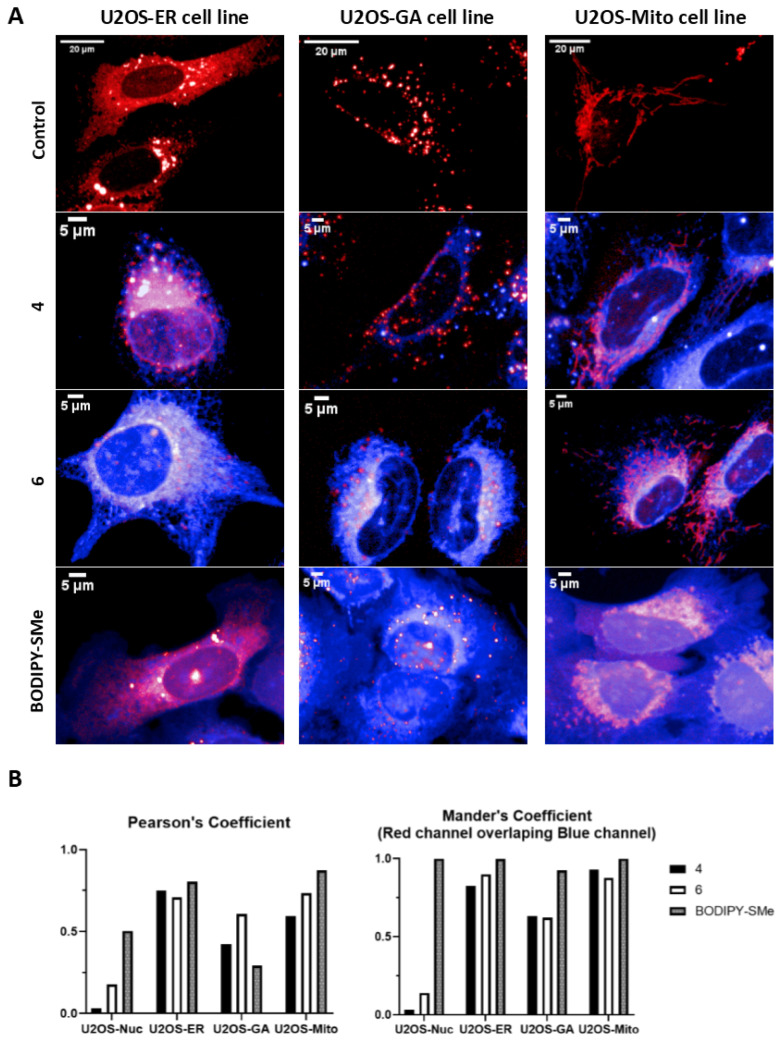
Live cell imaging and colocalization experiments of active compounds (**4**, **6**) and **BODIPY-SMe** (panel **A**) and visualization of Pearson´s and Mander´s coefficient (panel **B**).

The group of six derivatives of BA and BODIPY was studied on the U2OS-Nuc cell line with the nucleus labelled by fluorescein protein mCherry. The functionalized BODIPY dyes (**BODIPY-CO_2_H** and **BODIPY-NH_2_**), as well as precursor **BODIPY-SMe**, were used as a control. All fluorescent microscopic images of this pilot experiment are shown in Figure S53. To achieve a better specificity of the staining, we have focused on the short incubation with the fluorescent conjugates. After short incubation (1 h), conjugates **4** and **6** out of this group of derivatives were localized in living cells, but only with the weak signal in the nucleus of the studied cell line (Figure 5B—Pearson’s and Mander’s coefficients). The functionalized BODIPY dyes were not detected in the U2OS-Nuc cell line and thus it is highly possible that cellular uptake of conjugates **4** and **6** is due to their groups on BA residue. Other studied derivatives of BA and BODIPY were not detected in living cells under our experimental conditions; however, it is possible that the signal can be observed at later intervals. **BODIPY-SMe** is reactive due to the 8-thiomethyl group and it was predicted to penetrate cell compartments; this was confirmed by fluorescent microscopy.

To further study the cellular localization of conjugates **4** and **6**, we decided to continue with fluorescent microscopy on cell lines with fluorescently labelled structures of mitochondria, endoplasmic reticulum, and Golgi apparatus, which are the most published targets of BA [29,30]. The results of these colocalization experiments are shown in Figure 5. Both conjugates demonstrated presence in multiple cellular structures. Pearson’s coefficient (Figure 5B) showed the highest correlation of signal in U2OS-ER cell line, and then in U2OS-Mito cell line and the lowest correlation was measured in U2OS-GA cell line. When we expressed colocalization by Mander’s coefficient (overlap of red channel compared to the blue channel), which is more specific for colocalization calculation of signal presented in multiple cellular structures, the obtained data showed both conjugates **4** and **6** almost perfectly label mitochondria and endoplasmic reticulum. The lowest colocalization signal was again detected in the U2OS-GA cell line. **BODIPY-SMe** was used based on the data from the pilot experiment as a positive control with perfect colocalization in all studied cell lines. Images with entire microscopic fields are shown in Figures S54–S56.

To conclude our results from fluorescent microscopy study of six derivatives of BA and BODIPY, only conjugates **4** and **6** are detected in living cells under our experimental conditions (1 h following the treatment). Furthermore, we were able to almost perfectly colocalize both conjugates with cellular structures as endoplasmic reticulum and mitochondria, which is in agreement with data published in the past [21]. Compound **4** has, in its structure, fluorophore attached to the carboxyl group at the C-28 position, thus it is more similar to the pristine structure of BA and has low cytotoxicity (close to free BA). Conversely, compound **6** contains a conjugated amine at the C-28 position and the fluorophore is attached to the hydroxyl group at the C-3 position of BA. Its cytotoxicity is markedly more pronounced than in the case of substance **4**. It is clear that the “polar head” of the molecule is responsible for cytotoxicity. Moreover, this moiety can be used for the intracellular targeted delivery, or organelle/mitochondrion targeting, as is described by the previous research works [43,44]. As the localization of both compounds is similar, it is likely that the direct target remained unchanged, but the effect of compound **6** was potentiated by the presence of free amine moiety in the molecule. The localization of the compounds in lipid rich compartments (mitochondria, endoplasmic reticulum) can also be explained by the lipid character of the BA and its analogues. The calculated values of lipophilicity (logP) of the substances are close to BA (Table S2). The acidity constants (pKa) are indicative and their reproducibility is difficult because, in comparison with BA, the compounds described here are mostly in the form of amide or ester derivatives.

### 3.5. Anti-HIV Activity

Bevirimat (3-*O*-(3′,3′-dimethylsuccinyl) betulinic acid) and its derivatives were shown to be maturation inhibitors of HIV-1 [45,46,47]. By binding to the CA-SP1 region of HIV-1 Gag polyprotein, bevirimat prevents HIV-1 protease-mediated release of C-terminal part of CA from a spacer peptide 1 (SP1) [48]. This results in a block of the final step of virus maturation and subsequently abolishes HIV-1 infectivity. An atomic model of HIV-1 CA-SP1 suggested that this inhibitor stabilizes the CA-SP1 structure, thus preventing the proteolytic cleavage [49]. Although bevirimat is a potent inhibitor of HIV-1 maturation, its clinical development was discontinued in 2010 due to the bevirimat resistance caused by Gag SP1 natural polymorphism (Q6, V7 and T8) [50,51,52]. However, bevirimat derivatives with modification at the C-28 position seem to overcome the problem with HIV-1 resistance [53,54]. Here, using VSV-G pseudotyped HIV-1 particles, we tested the effect of 17 BA derivatives on HIV-1 maturation and infectivity. The 50% cytotoxic concentration (IC_50_) of the compounds was first evaluated by Resazurin assay. Two of the tested compounds, **3** and **14**, were highly toxic to HEK 293 cells at a concentration lower than 5 µM and significant cytotoxicity was also found for compound **6** (IC_50_ 12 µM) (Table 3).

**Figure 6:**
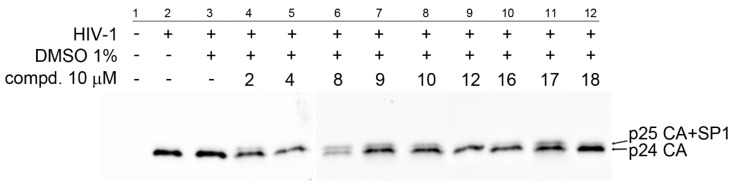
Effect of selected tested compounds on CA-SP1 processing of HIV-1 Gag polyprotein. HEK 293 cells produced HIV-1 particles pseudotyped with VSV-glycoproteins in the absence (lanes 2 and 3) or presence of selected tested compounds (lanes 4–12). At 48 h post-transfection, VSV-G pseudotyped HIV-1 viruses released from the HEK 293 cells were analysed by Western blot using an anti-HIV-1 CA antibody (duplicate of blot shown in Figure S57).

Apart from these three cytotoxic compounds, 14 fewer toxic compounds were used in the HIV-1 single-round infectivity assay. HIV-1 particles pseudotyped with VSV glycoproteins were produced in HEK 293 cells in the presence of tested compounds. At 48 h post-transfection, the content of HIV-1 capsid (CA) protein from the culture media was quantified by ELISA and normalized amounts of VSV-G pseudotyped HIV-1 viruses were used to infect fresh HEK 293 cells. At 48 h post-infection, the HIV-1 infectivity was determined by quantification of GFP-producing cells by flow cytometry. The 50% infection inhibition (IC_50i_) was defined as the concentration of the compound that reduced the HIV-1 infectivity by 50% compared to the untreated controls (Table 3). The compounds **1**, **7**, **13**, **15** and **5** did not exhibit any potent anti-HIV-1 activity (data not shown). Conversely, compounds **2**, **4**, **9** and **12** inhibited anti-HIV-1 activity with IC_50i_ from 11.7 to 44.1 µM. The compounds **8**, **10**, **16**, **17** and **18** inhibited HIV-1 with IC_50i_ below 10 µM (Table 3). To analyse whether these bevirimat derivatives also act as maturation inhibitors of CA-SP1 cleavage, the HIV-1 virions released from the HEK 293 cells treated with the selected compounds (**2**, **4**, **8**, **9**, **10**, **12**, **16**, **17** and **18**) were analysed by Western blot using anti-HIV-1 CA antibody (Figure 6).

Only completely processed p24 CA of molecular weight of 24 kDa was identified in the viruses formed in the presence of compounds **4** and **12**. However, in the samples treated with compounds **2**, **8**, **9**, **10**, **16**, **17** and **18**, we identified not only fully processed p24 CA, but also p25 CA-SP1 protein. This observation suggests a similar mechanism of inhibition as described for bevirimat, i.e., the block of the final step of HIV-1 maturation.

## 4. Conclusions

This study describes the synthesis and biological evaluation of 17 betulinic acid derivatives. The biological profiling revealed that BA derivatives **3** and **14** with modification at C-28 show increased cytotoxicity. However, the cytotoxicity was not specifically directed against cancer cell lines and was not associated with cell cycle arrest. The most effective compounds with sub-micromolar IC_50_ values **3** and **14** possess a hydroxyl group at C-3, whereas structures with a succinyl hemiester group displayed medium cytotoxicity or were inactive. The study introduced six original structures with BODIPY moiety linked to the lupane skeleton. BODIPY conjugates **4**, **5**, **15** and **18** showed low or no cytotoxic activity. In contrast, BODIPY derivative **6** induced strong and derivative **10** medium cytotoxicity in the entire cell line panel, although they do not share any similar substituents at positions C-3 and C-28. The cellular localization of BODIPY conjugates was further studied in U2OS cells using fluorescent microscopy. Fluorescent derivatives **4** and **6** colocalized with endoplasmic reticulum and mitochondria, which is in agreement with previous studies showing interaction with the processes and proteins localized in these organelles [55,56]. Uncoupling of the mitochondrial respiration, followed by radical burst and mitochondrial membrane disruption, is one of the well-described effects of betulin and betulinic acid [57,58,59,60]. Thus, we believe that reliable tools to study the derivatives of BA on living cells were established. The anti-HIV-1 activity showed that compounds **2**, **8**, **9**, **10**, **16** and **18** with IC_50i_ lower than 10 μM did not fully process the p24 CA and p25 CA-SP1 proteins, suggesting a similar mechanism of inhibition as described for bevirimat.

## Figures and Tables

**Table 1:** Summary of cytotoxic activities (IC_50_, μM).

Compound Cell Line ^a^	BA	BT	1	2	3	4	6	7	8	9	10	12	13	14	16	17	18
**CCRF-CEM**	>50	12.82	8.98	8.14	0.21	23.65	1.55	>50	8.18	22.48	2.92	9.62	>50	0.29	>50	5.76	8.61
**CEM-DNR**	23.05	22.17	16.84	10.23	1.22	>50	11.53	>50	9.24	>50	7.00	>50	4.76	0.35	>50	>50	>50
**K562**	>50	23.60	>50	25.99	0.90	>50	5.25	>50	19.18	>50	23.19	>50	5.09	0.40	47.90	>50	>50
**K562-Tax**	>50	22.03	10.58	15.94	0.37	>50	31.80	>50	13.30	>50	12.21	>50	8.77	0.52	>50	>50	>50
**A549**	22.68	23.06	>50	22.87	1.80	>50	6.65	21.29	18.84	>50	13.55	>50	5.15	1.26	44.93	>50	47.80
**HCT116**	>50	14.17	>50	19.40	0.82	>50	3.85	>50	13.21	>50	7.92	>50	6.02	0.39	46.63	>50	30.84
**HCT116p53−/−**	>50	18.20	>50	29.24	0.44	>50	3.39	>50	21.56	>50	8.80	>50	4.99	0.44	44.76	>50	45.50
**U2OS**	29.69	27.63	29.04	22.22	0.89	>50	5.00	18.39	17.16	>50	12.38	>50	4.16	0.42	44.62	44.58	>50
**MRC-5**	>50	>50	>50	24.19	2.59	>50	8.07	17.58	23.14	>50	14.12	>50	5.18	1.58	44.61	>50	>50
**BJ**	>50	>50	>50	25.33	1.91	>50	8.37	20.69	21.54	>50	15.49	>50	5.36	1.59	47.63	>50	>50

^a^ Cytotoxic activity was determined by MTS assay following 3-day incubation. Values represent means of IC_50_ from three independent experiments with SD ranging from 10–25% of the average values. Tested cell lines: CCRF-CEM (childhood T acute lymphoblastic leukaemia), CEM-DNR (CCRF-CEM daunorubicin resistant), K562 (chronic myelogenous leukaemia), K562-Tax (K562 paclitaxel-resistant), A549 (lung adenocarcinoma), HCT116 (colorectal cancer), HCT116p53−/− (null p53 gene), and U2OS (osteosarcoma). Normal human cell lines: MRC-5 and BJ (normal cycling fibroblasts). BA, betulinic acid; BT, bevirimat.

**Table 2:** Effect of cytotoxic compounds on cell cycle, apoptosis and DNA/RNA synthesis in CCRF-CEM lymphoblasts (% of positive cells).

Compound	<G1	G0/G1	S	G2/M	pH3^Ser10 *a*^	BrDU *^b^*	BrU *^c^*
control	2.20	40.77	37.64	21.60	1.77	37.22	40.77
**1** 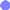	1.36	37.03	44.13	18.84	1.53	36.54	52.69
**1** 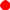	1.93	42.33	35.87	21.80	1.71	37.99	44.47
**2** 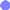	4.33	39.88	46.65	13.47	1.33	52.07	28.60
**2** 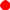	41.17	23.94	56.21	19.85	0.58	22.75	4.05
**3** 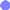	1.30	35.07	36.54	28.40	2.04	41.88	50.56
**3** 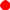	3.53	31.73	41.18	27.09	0.16	2.06	0.40
**6** 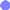	2.23	33.39	49.74	16.87	1.50	44.97	42.25
**6** 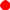	5.24	37.16	39.52	23.32	0.50	7.10	26.05
**8** 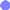	2.64	31.93	47.72	20.35	1.70	46.44	33.61
**8** 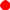	39.65	40.78	37.66	21.56	1.25	8.51	1.85
**10** 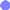	3.80	37.98	44.59	17.43	1.44	42.25	35.04
**10** 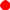	10.69	44.58	42.32	13.10	1.25	10.18	13.02
**12** 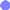	2.45	33.64	43.16	23.20	1.40	59.73	41.98
**12** 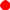	3.01	29.75	56.69	13.55	0.68	64.07	34.00
**14** 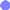	8.63	21.46	56.29	22.25	0.15	24.55	1.56
**14** 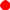	8.16	27.28	51.82	20.90	0.18	0.52	1.53
**17** 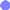	2.89	34.83	46.96	18.21	1.56	48.07	26.70
**17** 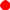	4.04	43.42	38.47	18.10	1.49	29.41	3.39
**18** 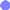	2.06	36.06	44.31	19.63	1.25	55.81	46.98
**18** 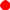	2.97	38.01	44.12	17.87	1.55	49.64	70.23

Flow cytometry analysis was used for quantification of cell cycle distribution and apoptotic cells with a concentration of compounds equal to 1 × IC_50_ (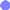) and 5 × IC_50_ (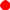) values. *^a^* phospho-Histone (Ser10); *^b^* 5-bromo-2-deoxyuridine; *^c^* BrU, 5-bromouridine.

**Table 3:** Cytotoxicity and anti-HIV-1 activity of the tested compounds ^a^.

Compd.	1	2	4	5	6	7	8	9	10	12	13	15	16	17	18
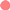 **IC_50_ [μM]**	>40	36.4	>40	>40	12.0	>40	37.8	>40	>40	>40	>40	>40	>40	>40	>40
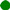 **IC_50i_ [μM]**	>50	11.7	44.1	>50	n.d.	>50	1.4	14.0	8.4	31.9	>50	>50	9.1	7.6	7.1

^a^ HEK 293 cells were grown in the presence or absence of tested compounds at a concentration ranging from 5 to 40 µM. The viability of the cells was determined by Resazurin assay 48 h later (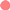). To determine the effect of the compounds on HIV-1 infectivity (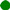), HEK 293 cells were transfected with the lentiviral vectors and treated with the tested compounds. The cells producing HIV-1 particles in the presence or absence of DMSO (at a final concentration of 1%) were used as controls. At 48 h post-transfection, the content of HIV-1 capsid (CA) protein from the culture media was quantified by ELISA and normalized amounts of VSV-G pseudotyped HIV-1 viruses were used to infect fresh HEK 293 cells. HIV-1 infectivity was determined 48 h later by quantification of GFP-producing cells by flow cytometry. The 50% infection inhibition (IC_50i_) was defined as the concentration of the compound that reduced the HIV-1 infectivity by 50% compared to the untreated controls.

## Supplementary Materials

The following are available online at https://www.mdpi.com/article/10.3390/biomedicines9091104/s1. Supplementary Figures S1–S52 and Table S1 document the analytical identification (NMR, HRMS, UV-Vis and fluorescence). Table S2: Calculated physical properties (pKa and logP) of the derivatives. Figures S53–S56 show supplementary pictures from fluorescent microscopy. Figure S57: Effect of selected tested compounds on CA-SP1 processing of HIV-1 Gag polyprotein (a duplicate of western blot showed in Figure 5. in the article).
