# Supplementary material for: Betulinic Acid Decorated with Polar Groups and Blue Emitting BODIPY Dye: Synthesis, Cytotoxicity, Cell-Cycle Analysis and Anti-HIV Profiling
Source: Biomedicines. 2021 Aug 28;9(9):1104. doi: 10.3390/biomedicines9091104 (PMC8472287; doi:10.3390/biomedicines9091104)
Supplement: Supplementary file 1 [file biomedicines-09-01104-s001.zip › biomedicines-1332342-supplementary.pdf]

Supplementary Information

Betulinic Acid Decorated with Polar Groups and Blue Emitting BODIPY Dye: Synthesis, Cytotoxicity, Cell-Cycle Analysis and Anti-HIV Profiling

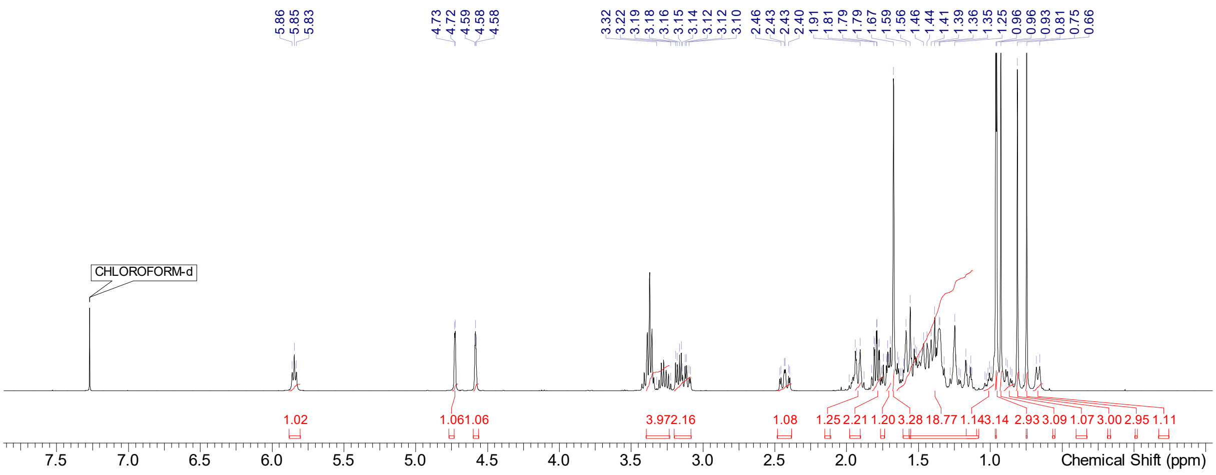

Figure S1. ^{1}H NMR of **1** in $CDCl_3$.

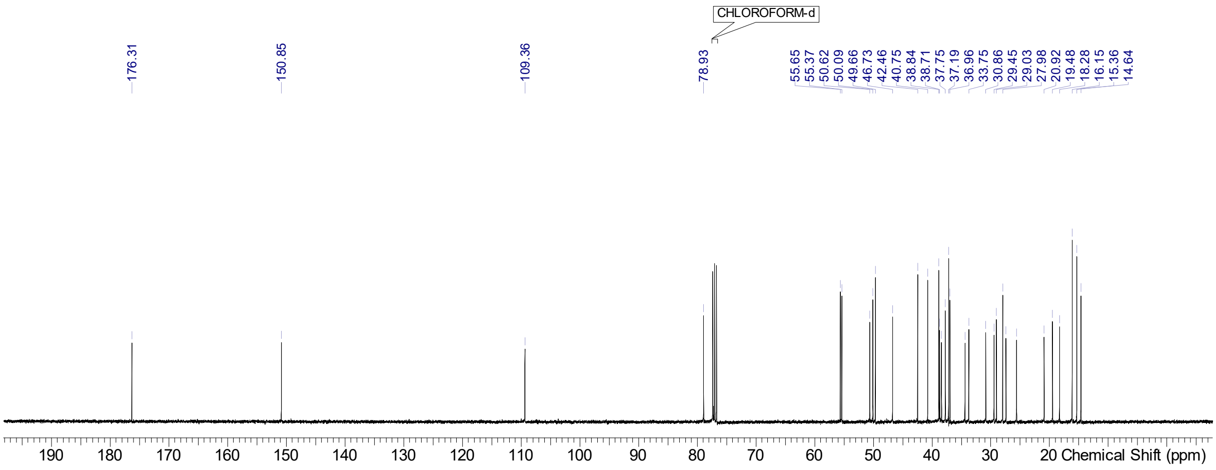

Figure S2. ^{13}C NMR of **1** in $CDCl_3$.

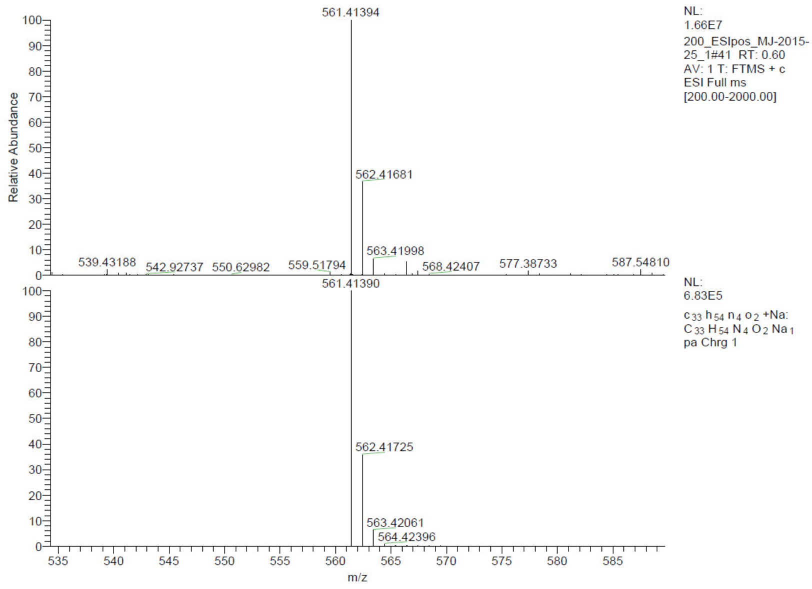

Figure S3. HRMS-ESI spectrum of **1**.

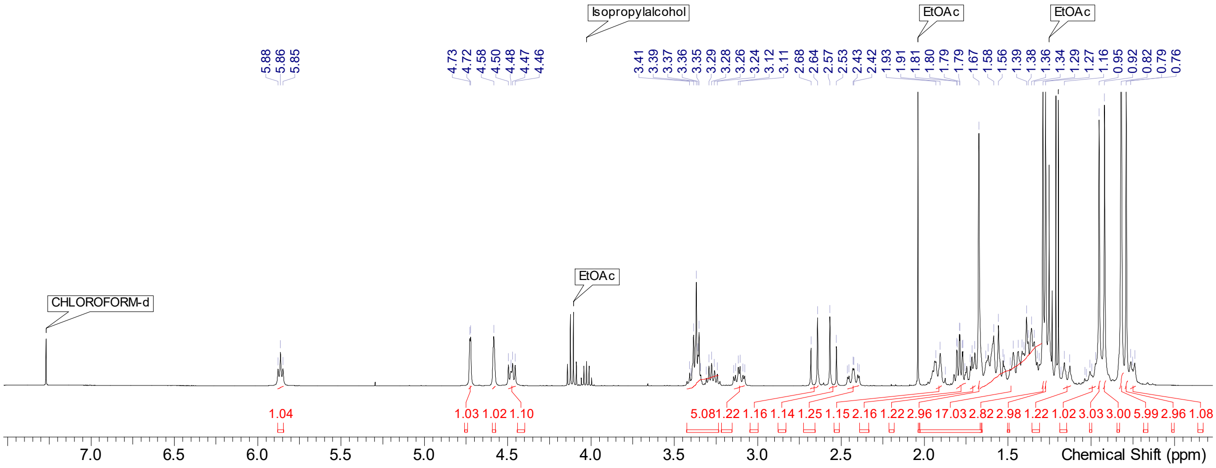

Figure S4. ^{1}H NMR of **2** in $CDCl_3$.

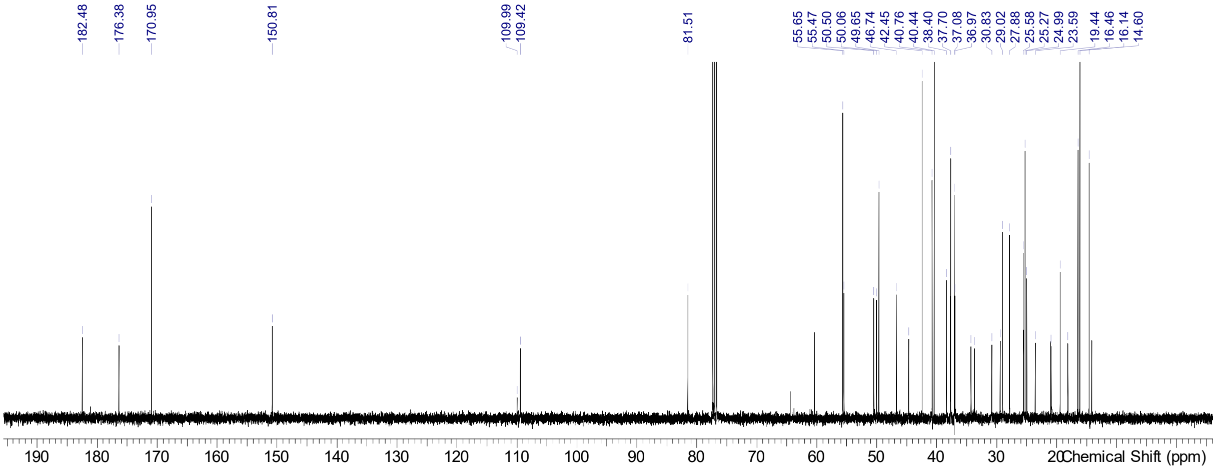

Figure S5. ^{13}C NMR of **2** in $CDCl_3$.

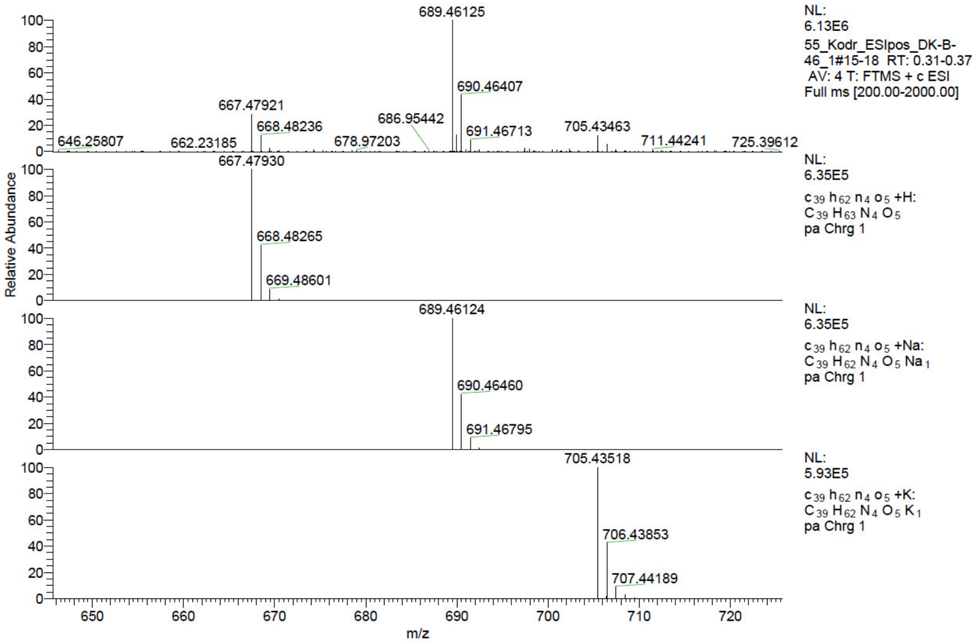

Figure S6. HRMS-ESI spectrum of **2.**

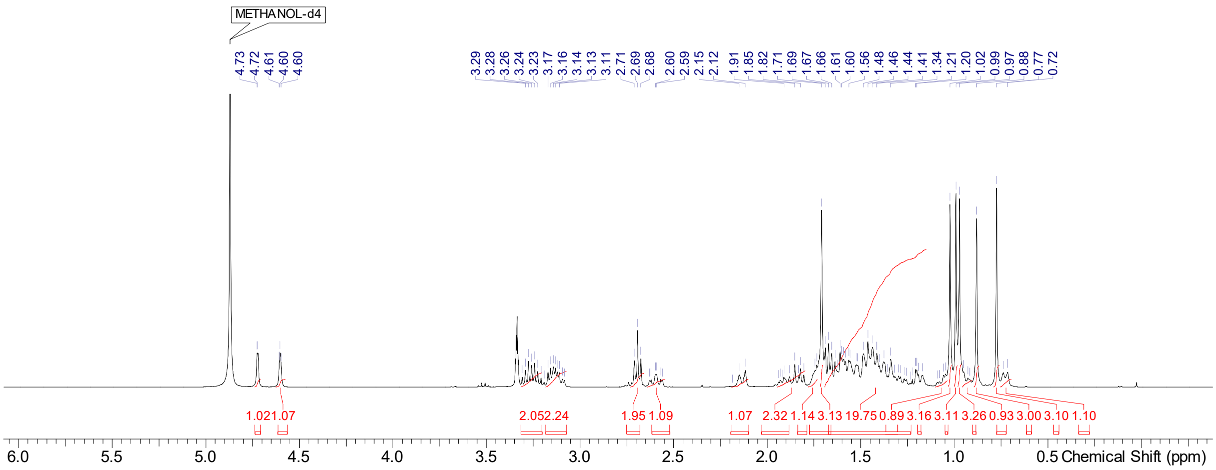

Figure S7. ^1H NMR of **3** in CD_3OD.

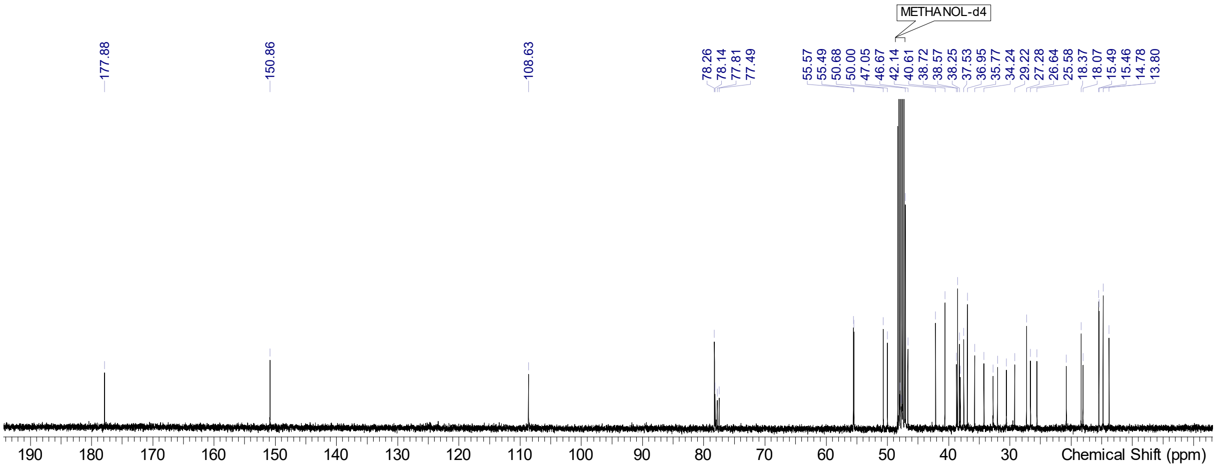

Figure S8. ^{13}C NMR of **3** in CD_3OD.

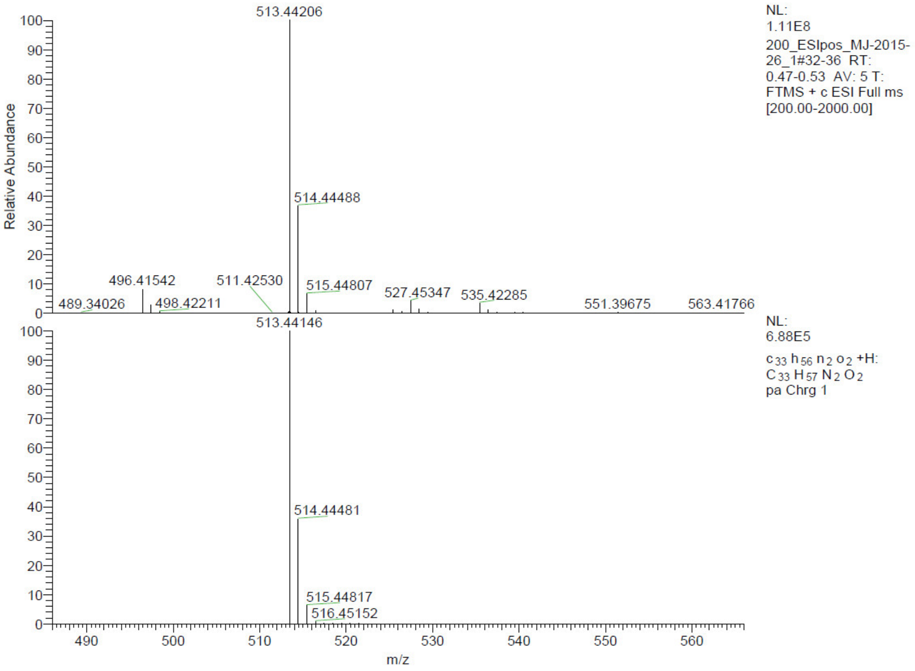

Figure S9. HRMS-ESI spectrum of **3.**

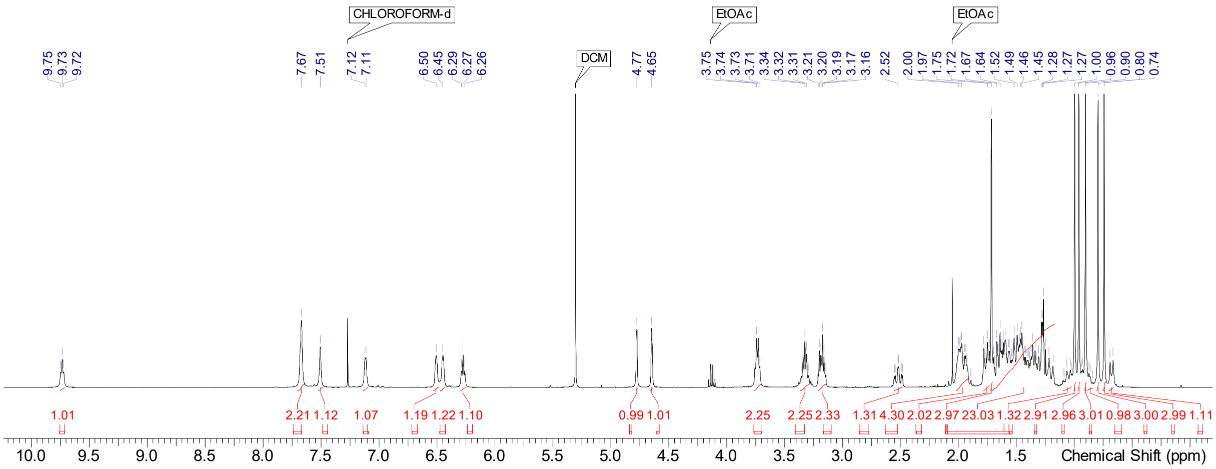

Figure S10. ^{1}H NMR of **4** in $CDCl_3$.

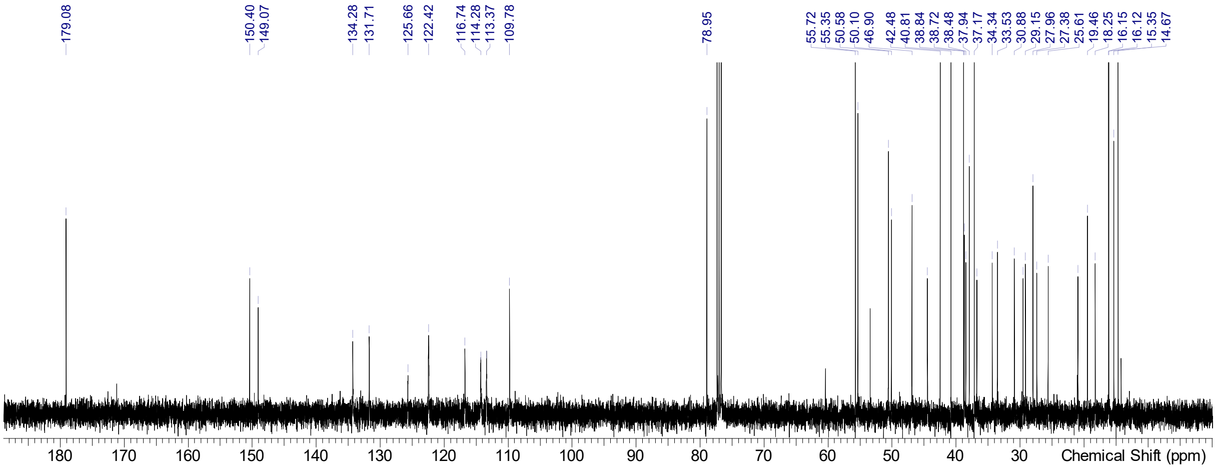

Figure S11. ^{13}C NMR of **4** in $CDCl_3$.

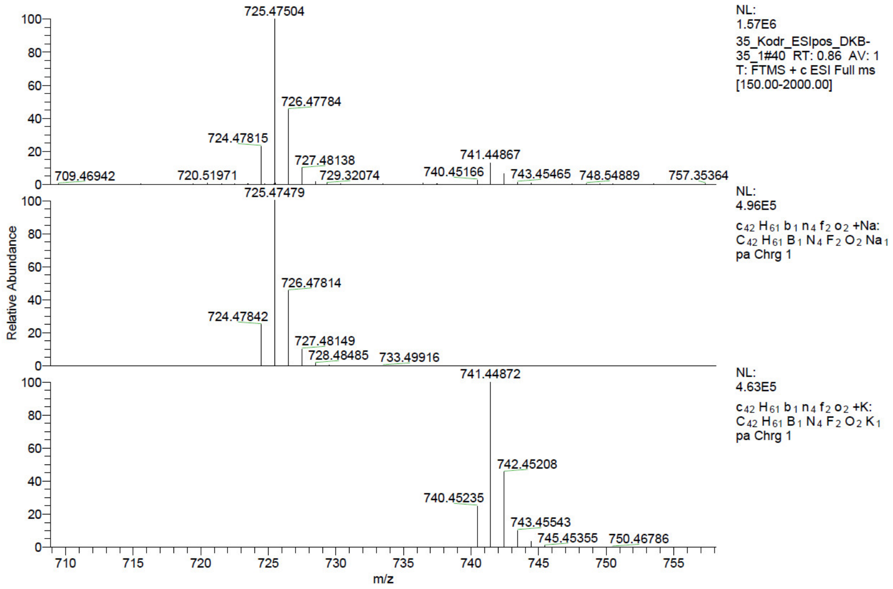

Figure S12. HRMS-ESI spectrum of **4.**

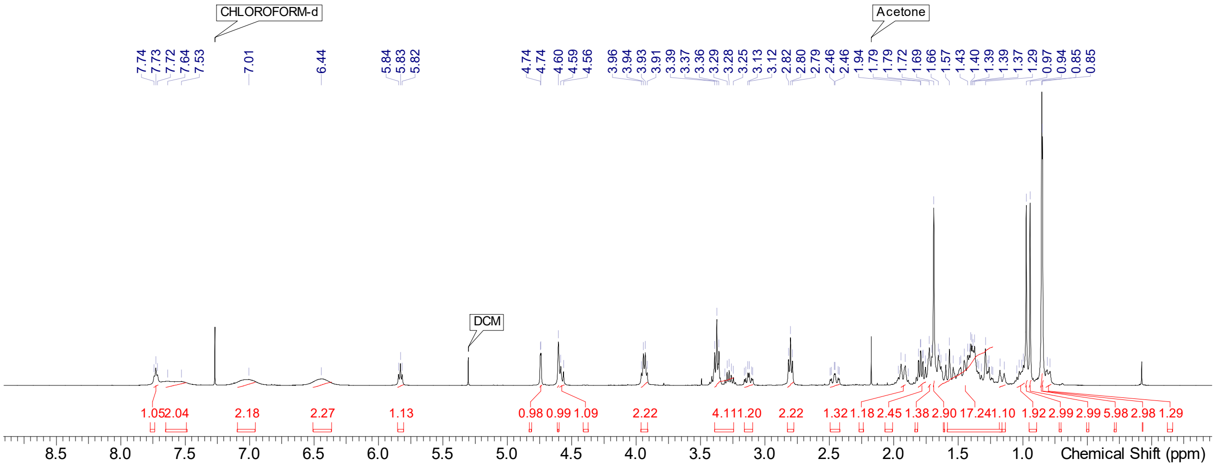

Figure S13. ^{1}H NMR of **5** in $CDCl_3$.

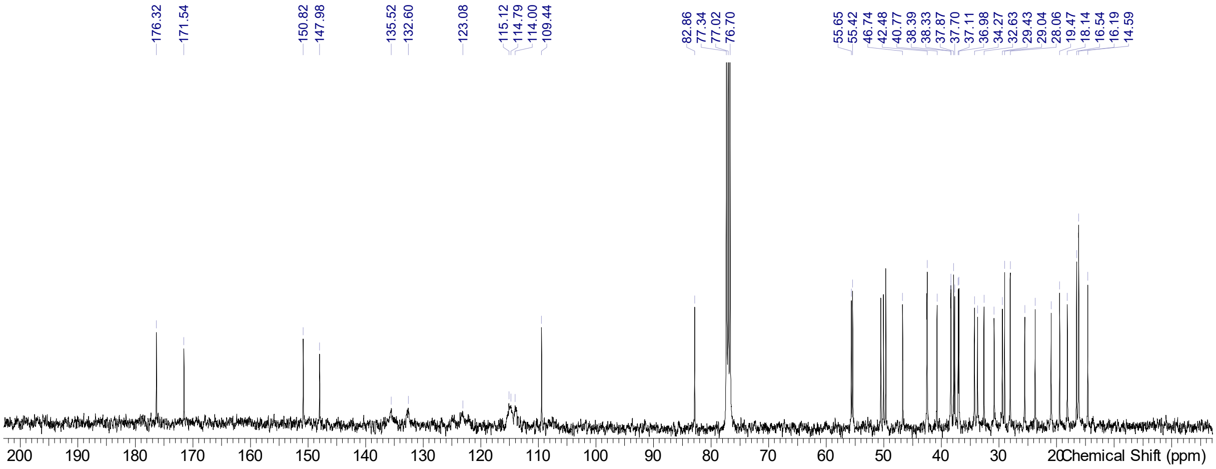

Figure S14. ^{13}C NMR of **5** in $CDCl_3$.

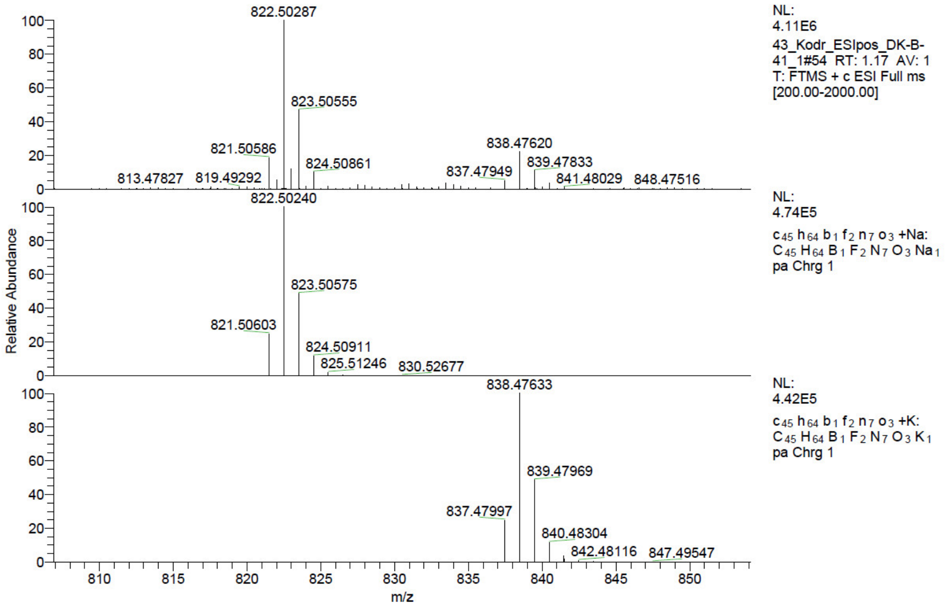

Figure S15. HRMS-ESI spectrum of **5.**

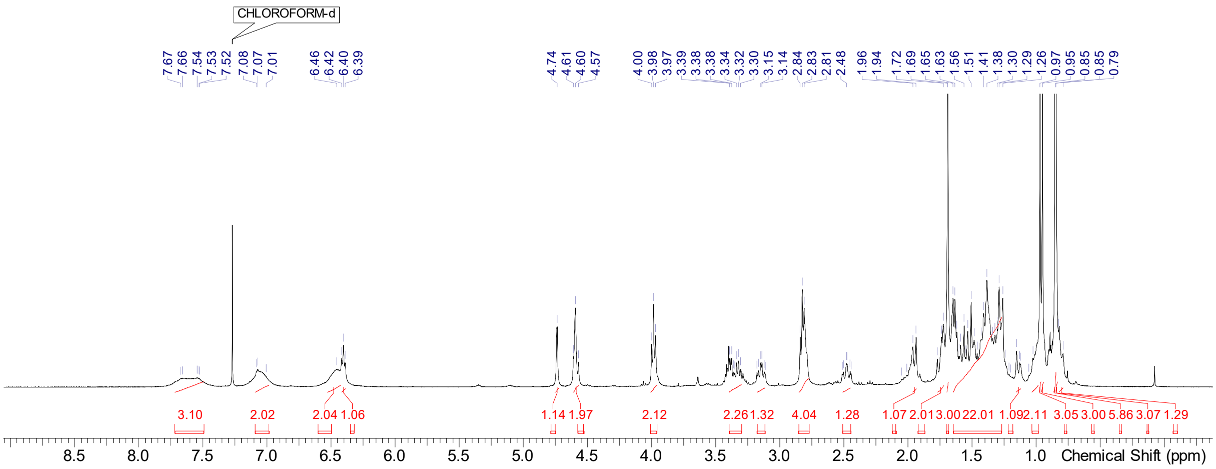

Figure S16. ^{1}H NMR of **6** in $CDCl_3$.

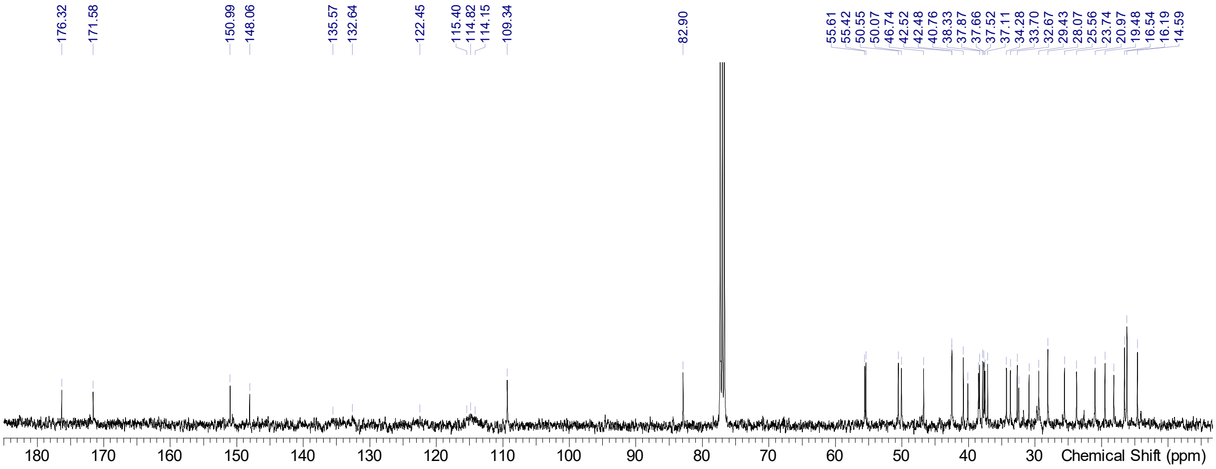

Figure S17. ^{13}C NMR of **6** in $CDCl_3$.

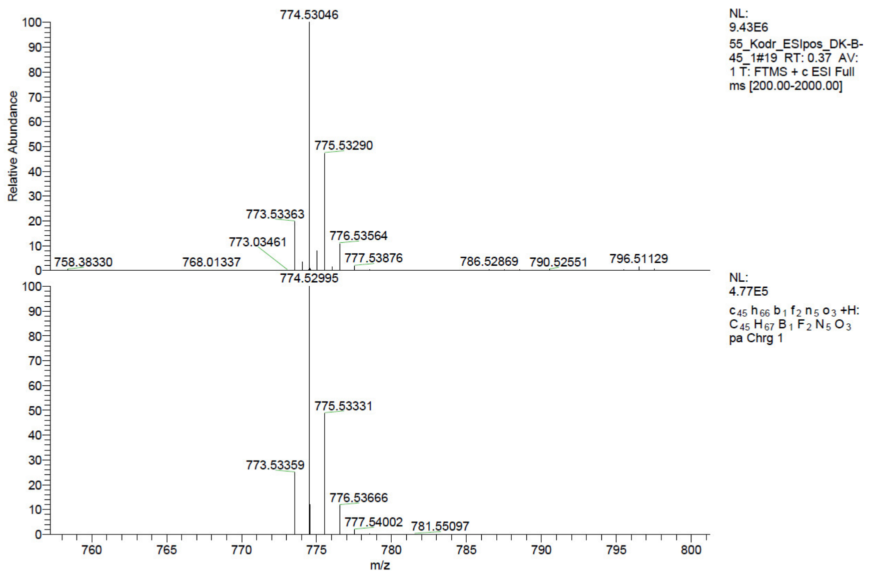

Figure S18. HRMS-ESI spectrum of **6.**

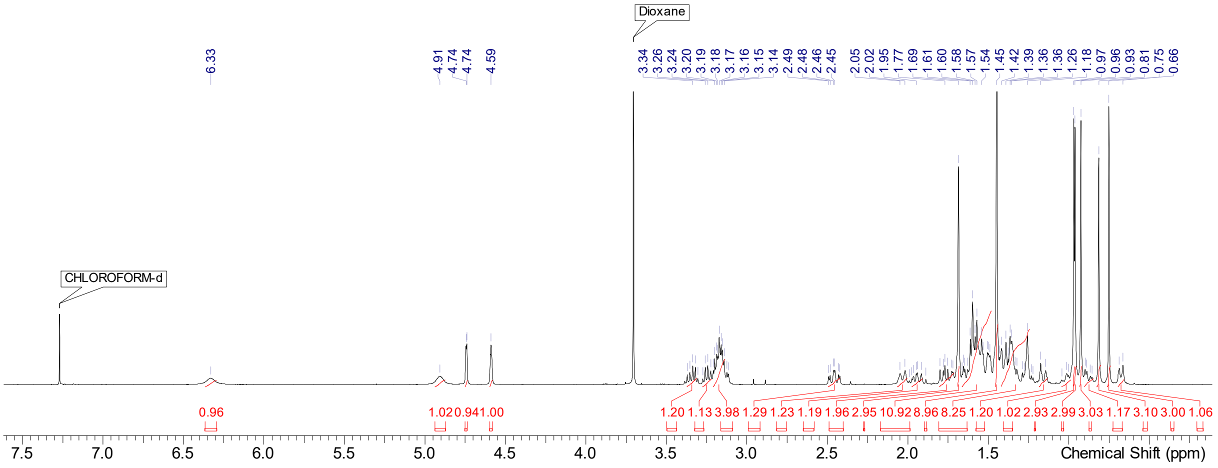

Figure S19. ^{1}H NMR of **7** in $CDCl_3$.

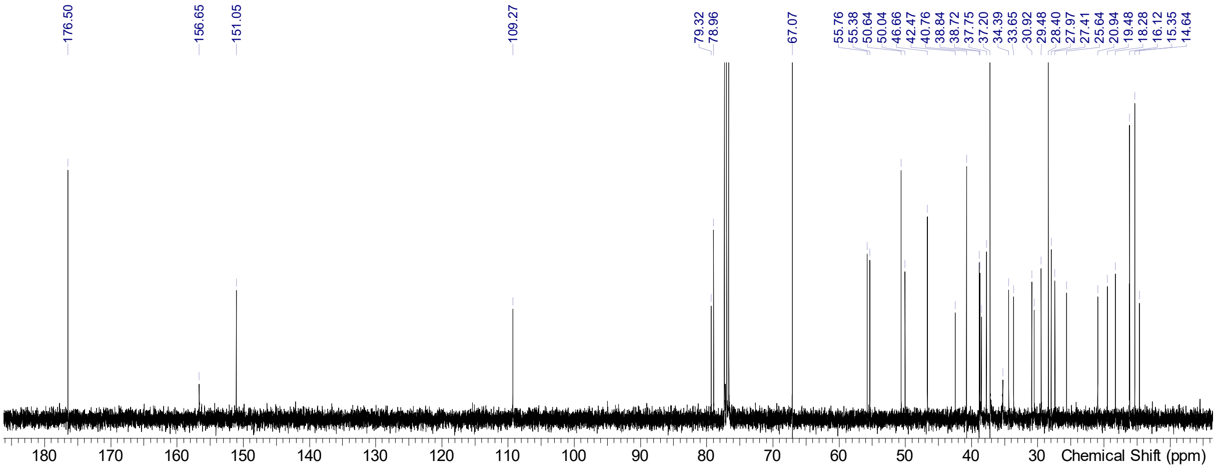

Figure S20. ^{13}C NMR of **7** in $CDCl_3$.

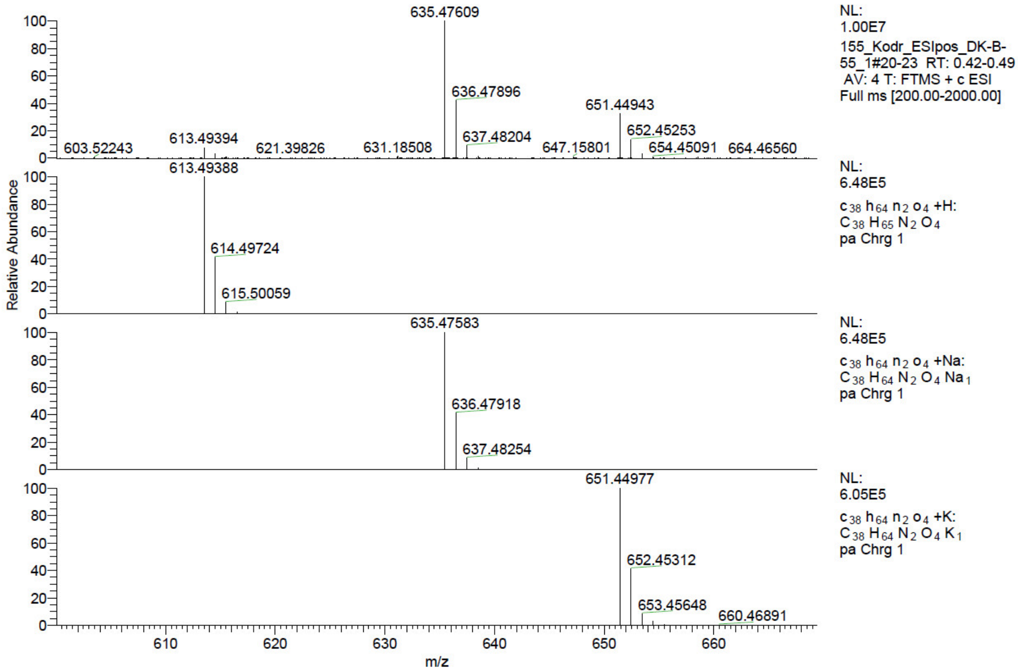

Figure S21. HRMS-ESI spectrum of **7.**

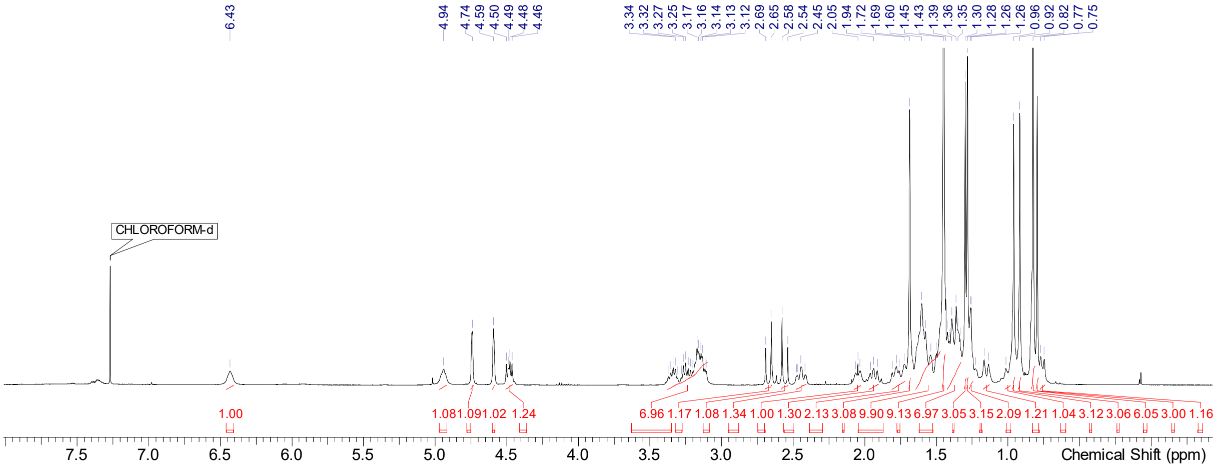

Figure S22. ^{1}H NMR of **8** in $CDCl_3$.

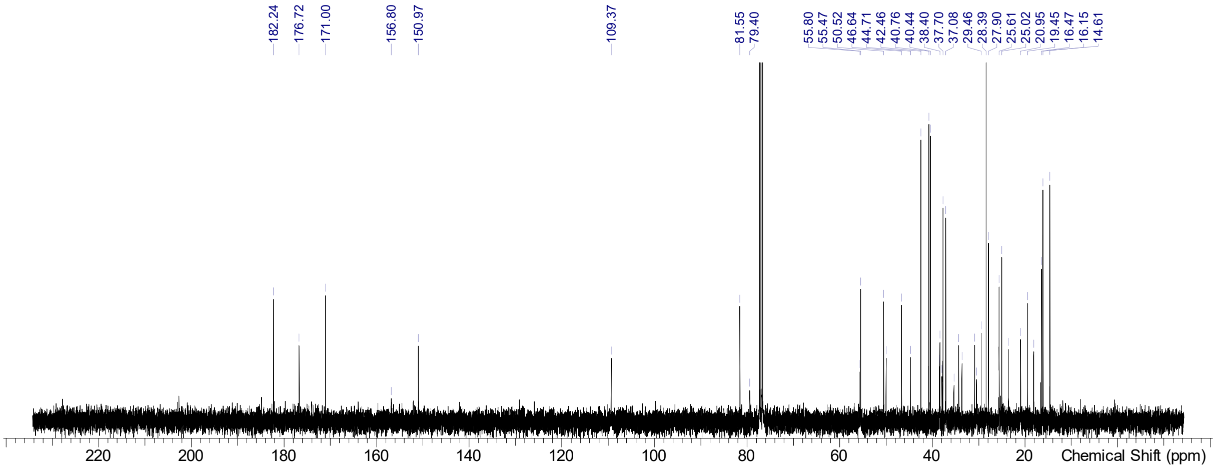

Figure S23. ^{13}C NMR of **8** in $CDCl_3$.

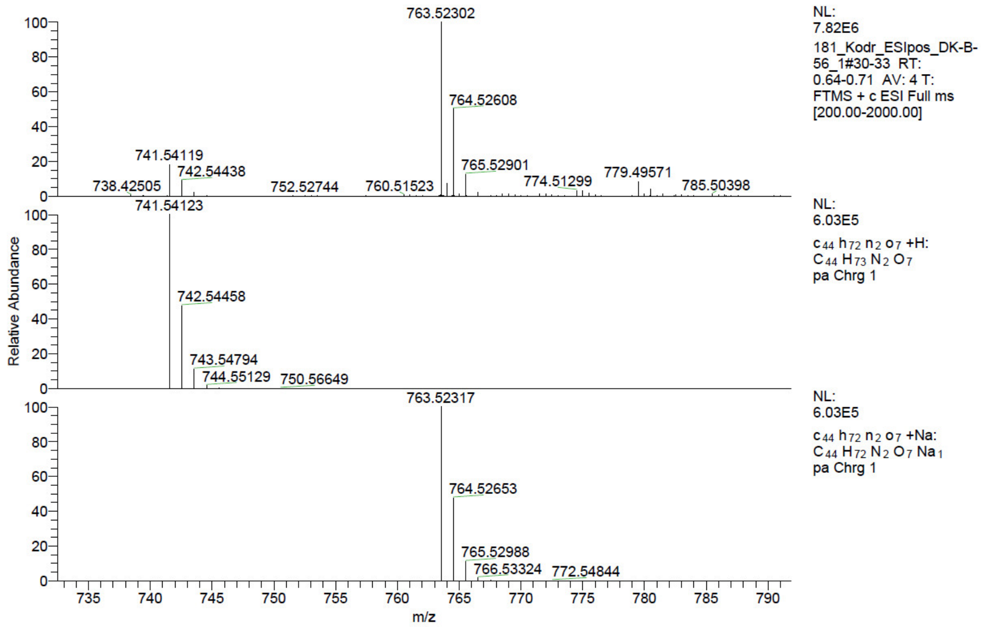

Figure S24. HRMS-ESI spectrum of **8.**

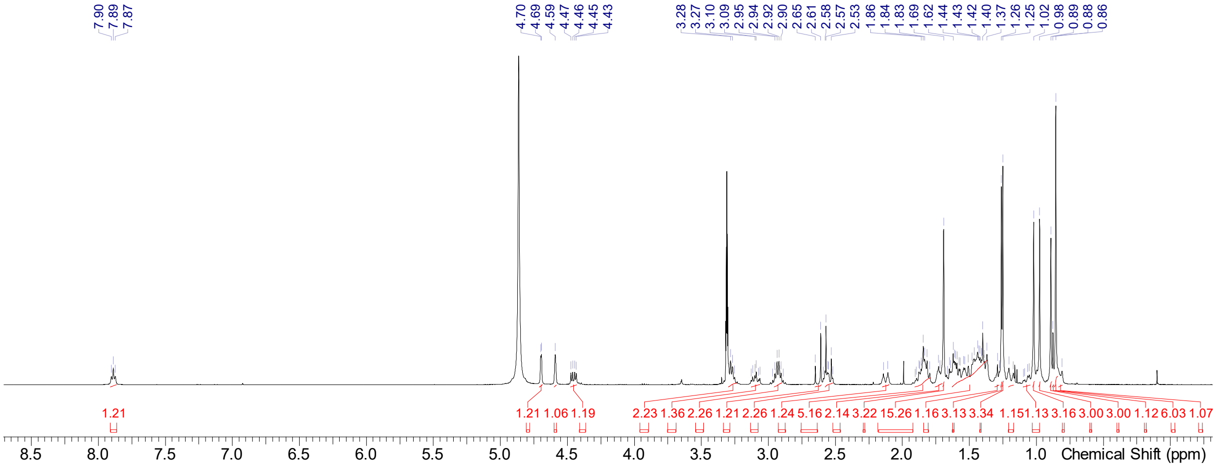

Figure S25. ^{1}H NMR of **9** in CD_3OD.

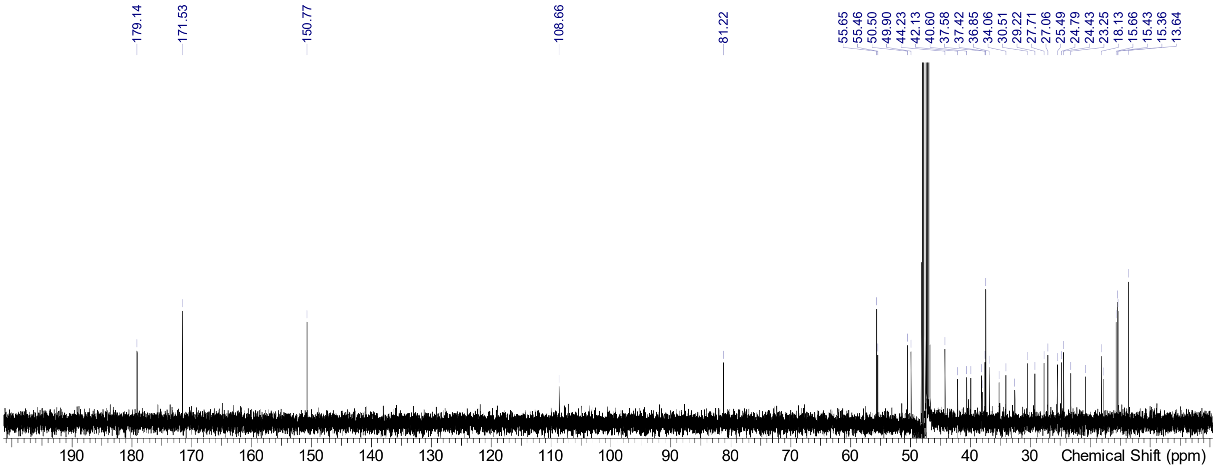

Figure S26. ^{13}C NMR of **9** in CD_3OD.

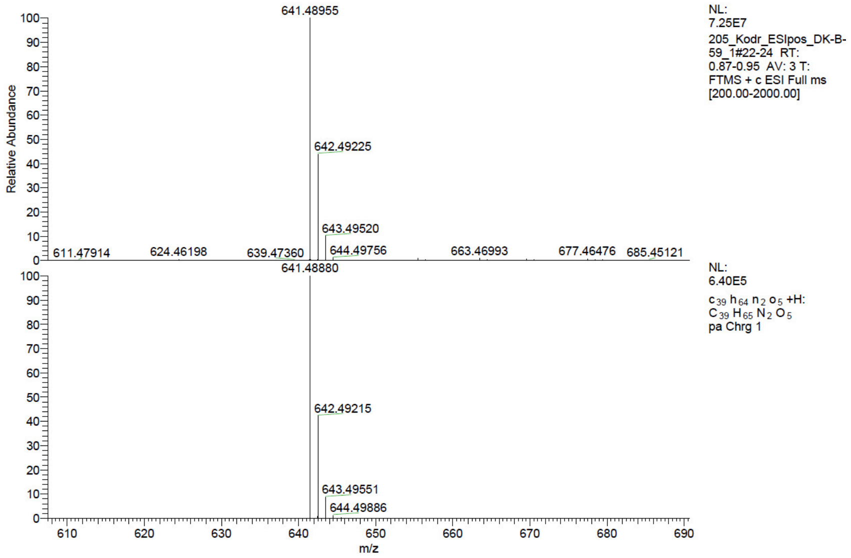

Figure S27. HRMS-ESI spectrum of **9.**

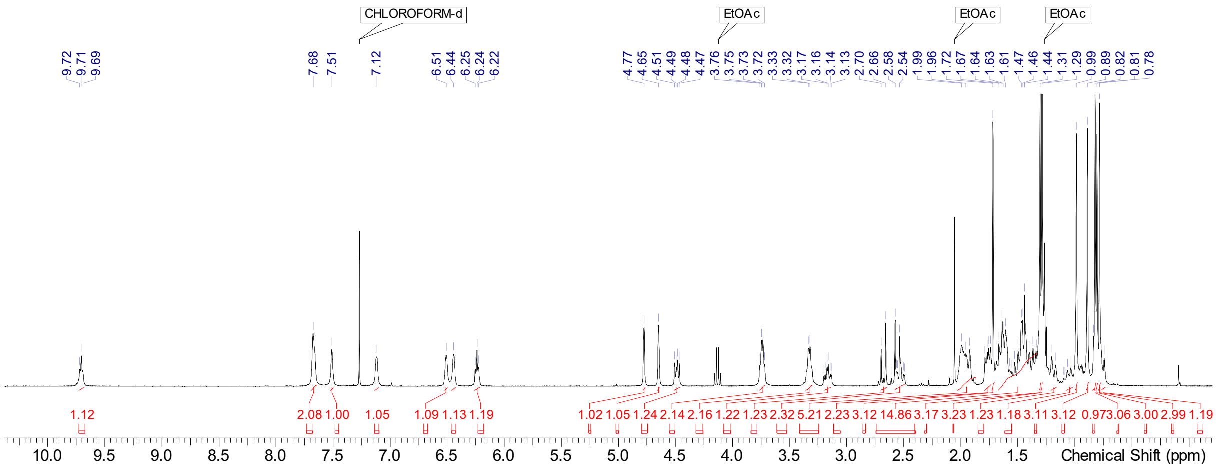

Figure S28. ^{1}H NMR of **10** in $CDCl_3$.

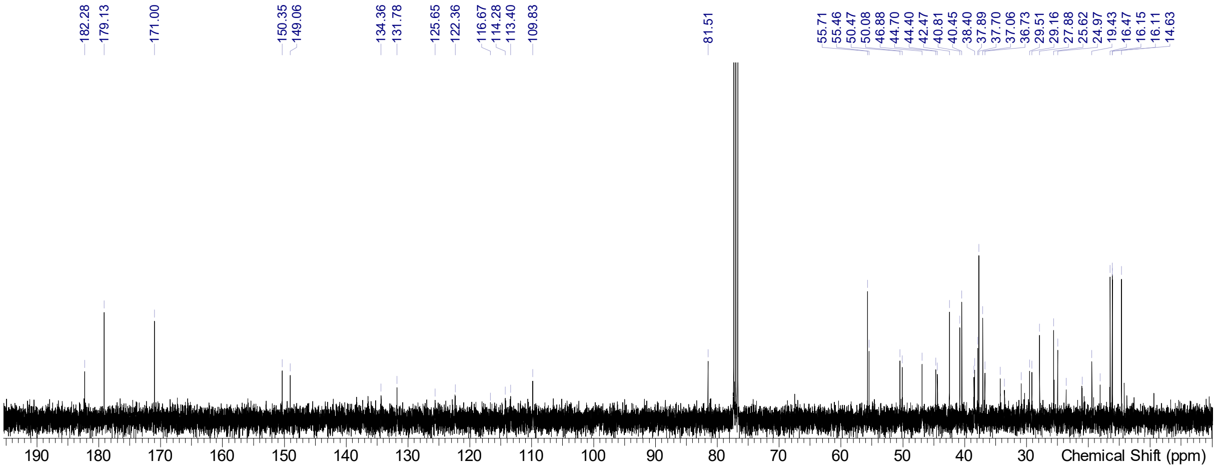

Figure S29. ^{13}C NMR of **10** in $CDCl_3$.

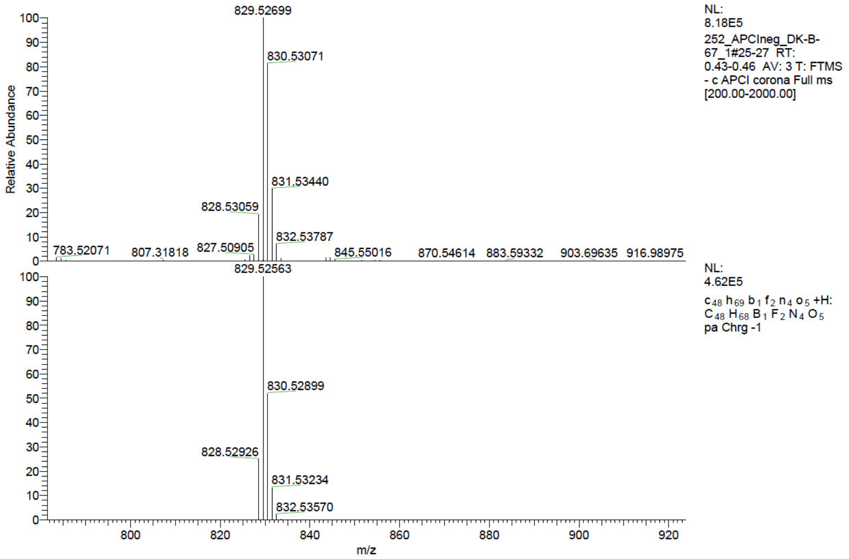

Figure S30. HRMS-APCI spectrum of **10**.

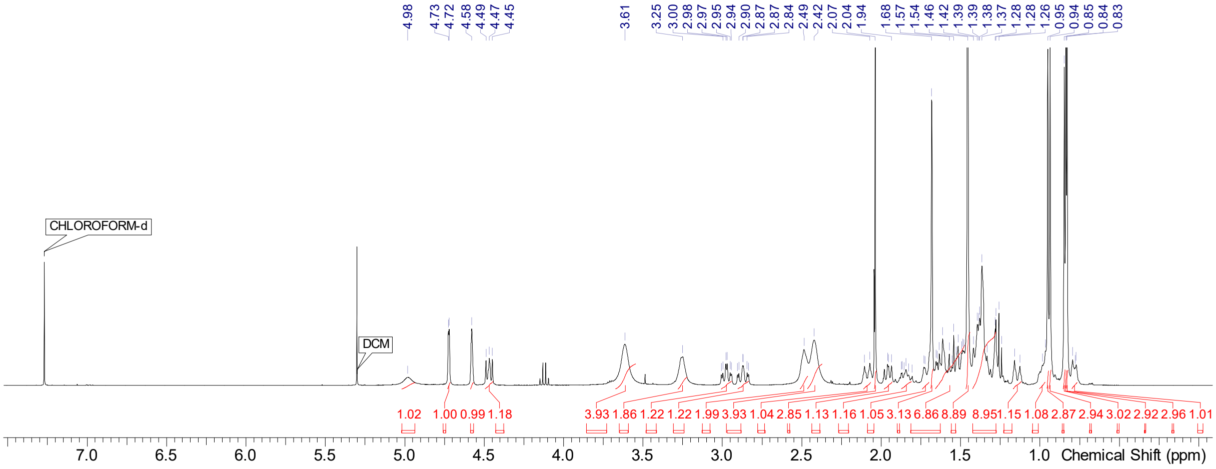

Figure S31. ^{1}H NMR of **12** in $CDCl_3$.

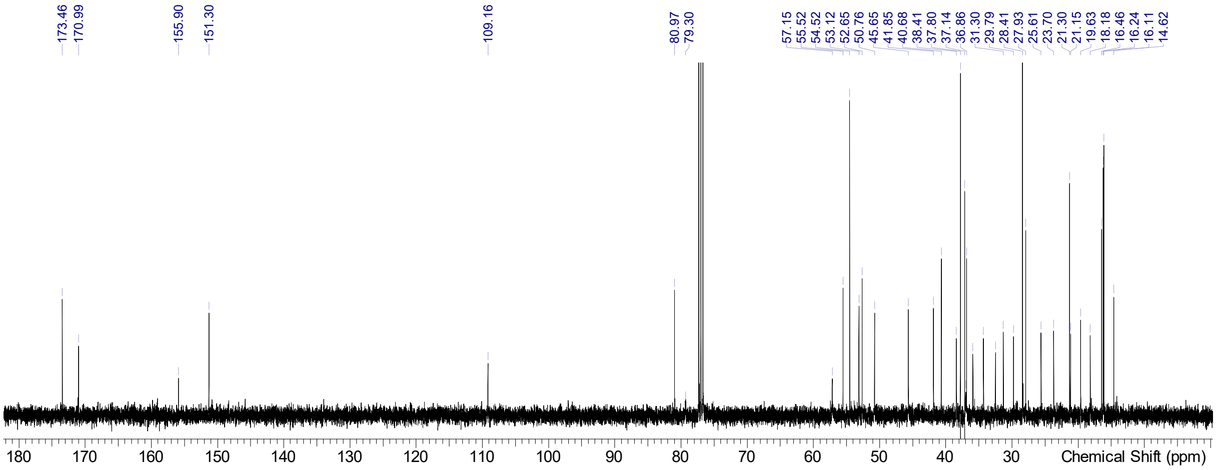

Figure S32. ^{13}C NMR of **12** in $CDCl_3$.

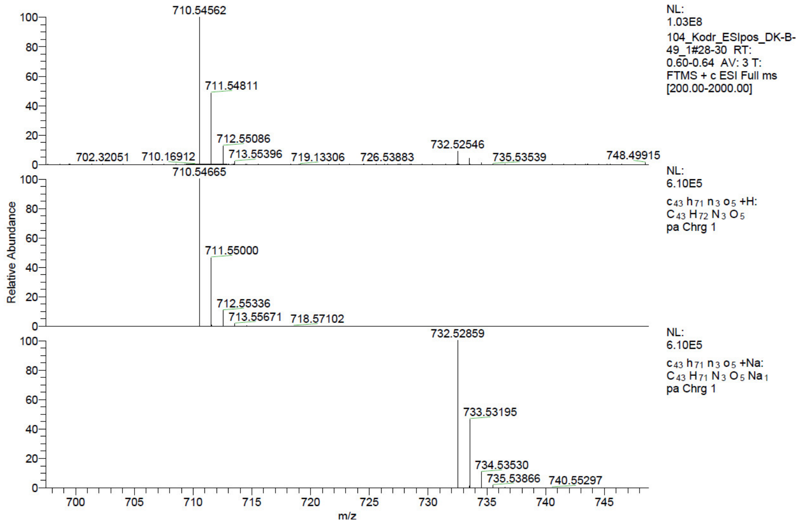

Figure S33. HRMS-ESI spectrum of **12.**

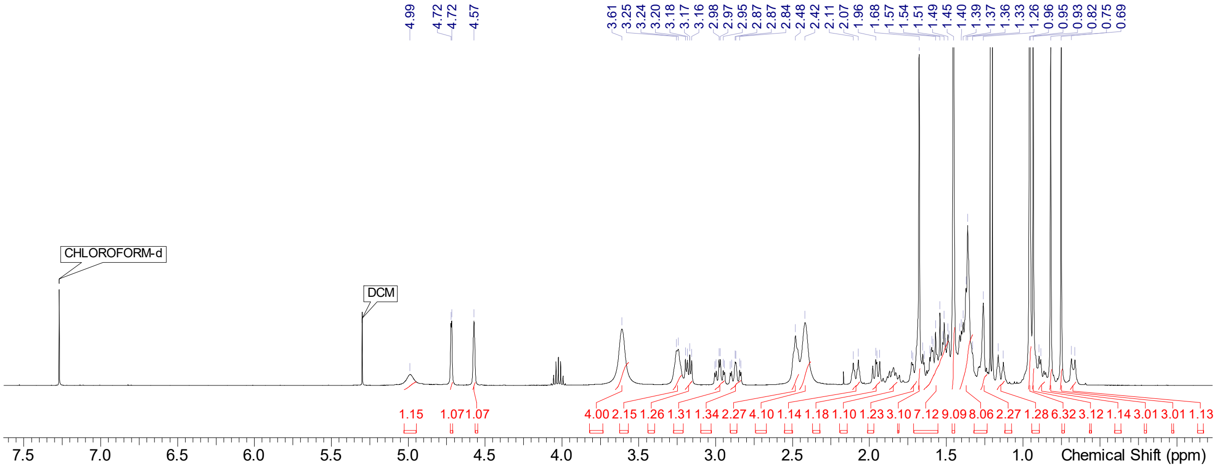

Figure S34. ^{1}H NMR of **13** in $CDCl_3$.

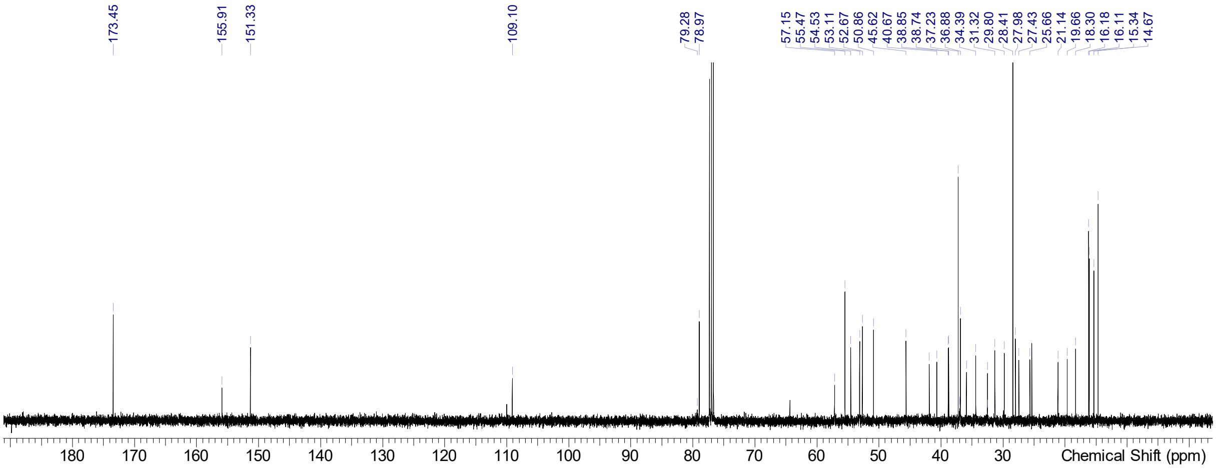

Figure S35. ^{13}C NMR of **13** in $CDCl_3$.

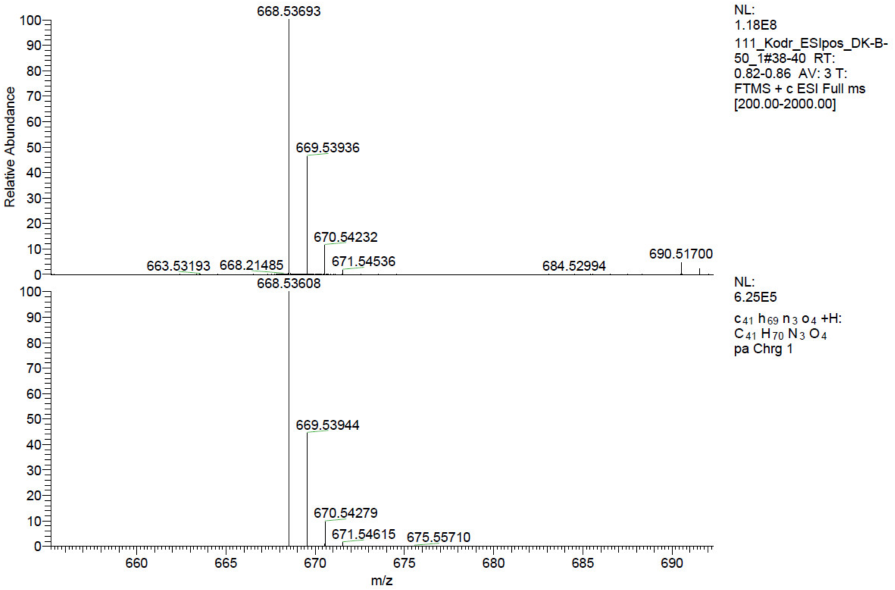

Figure S36. HRMS-ESI spectrum of **13.**

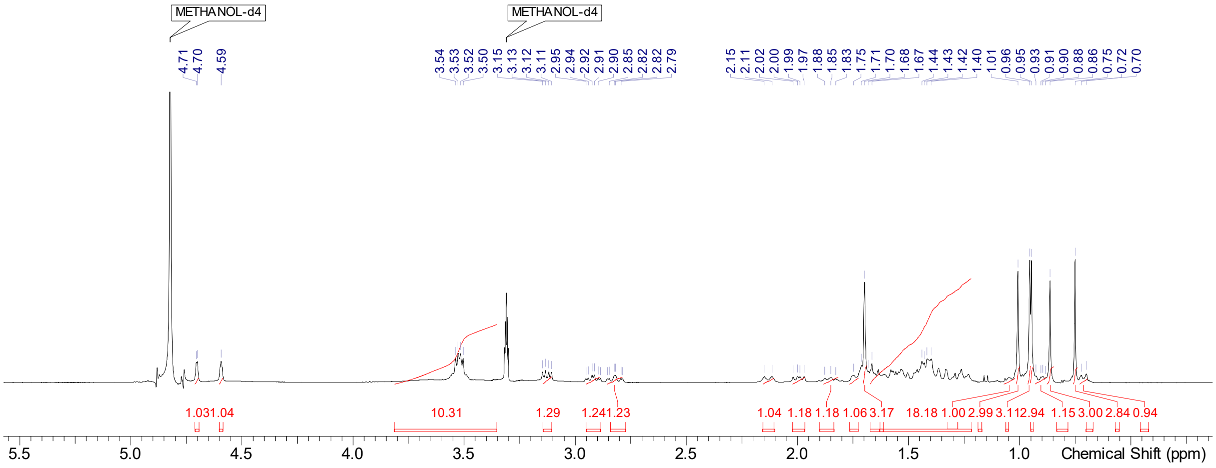

Figure S37. ^{1}H NMR of **14** in $CDCl_3$.

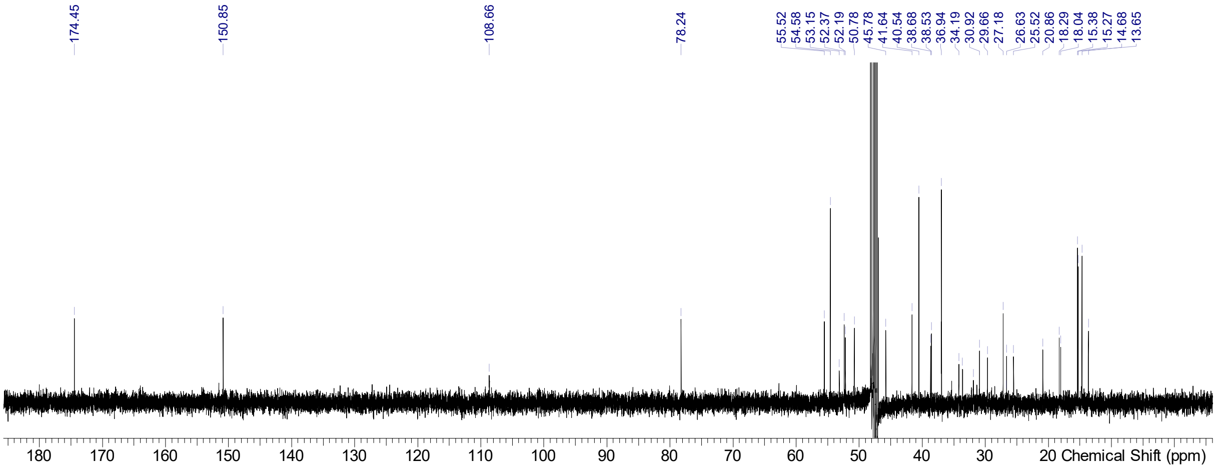

Figure S38. ^{13}C NMR of **14** in $CDCl_3$.

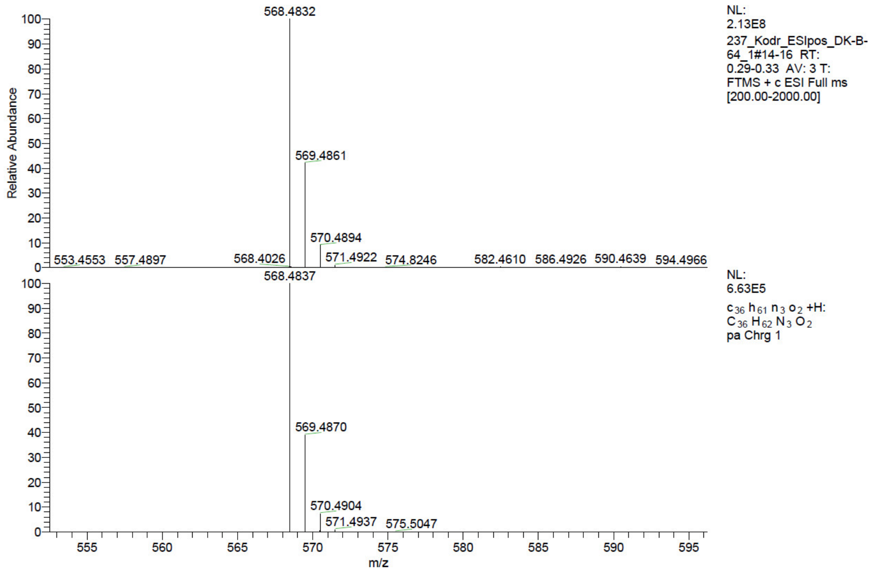

Figure S39. HRMS-ESI spectrum of **14.**

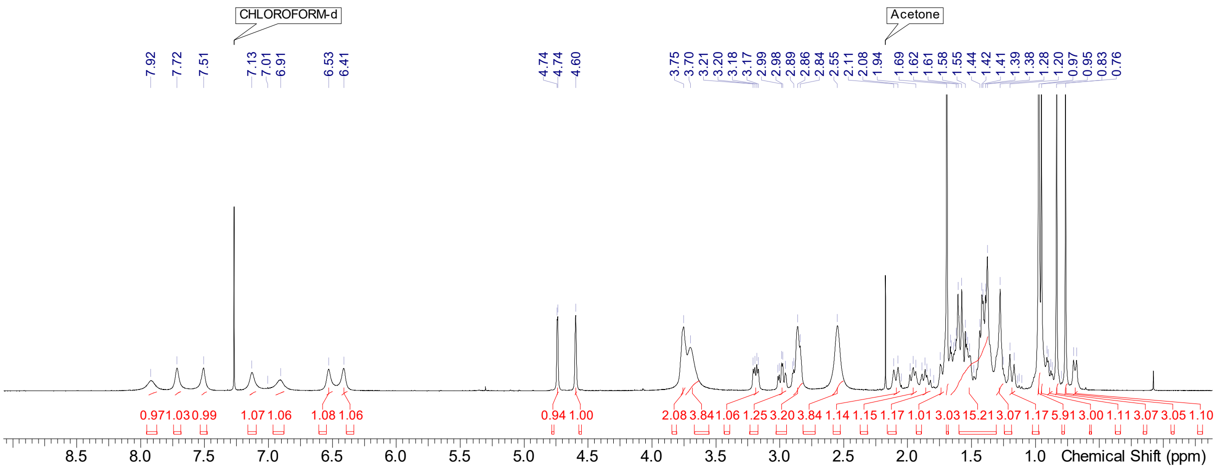

Figure S40. ^{1}H NMR of **15** in $CDCl_3$.

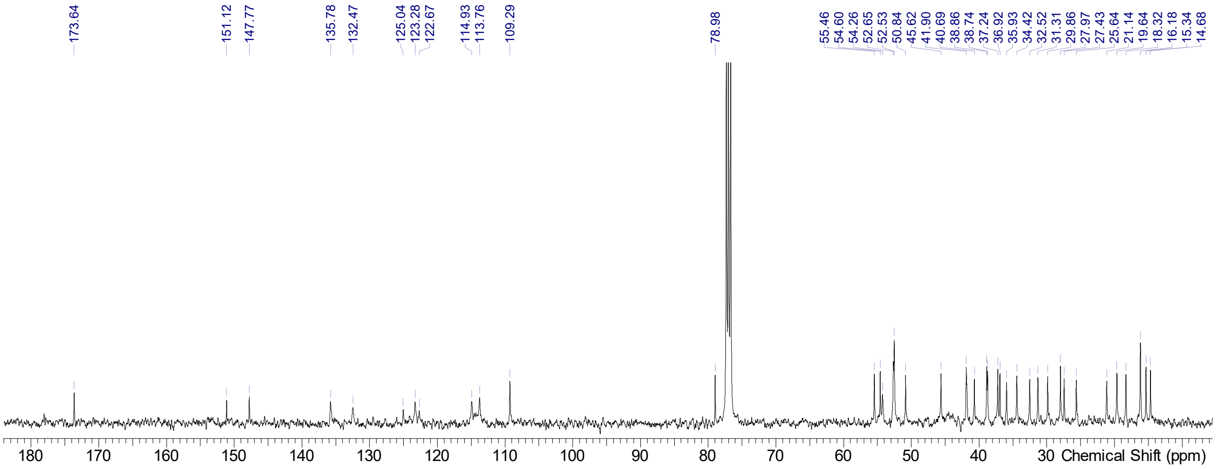

Figure S41. ^{13}C NMR of **15** in $CDCl_3$.

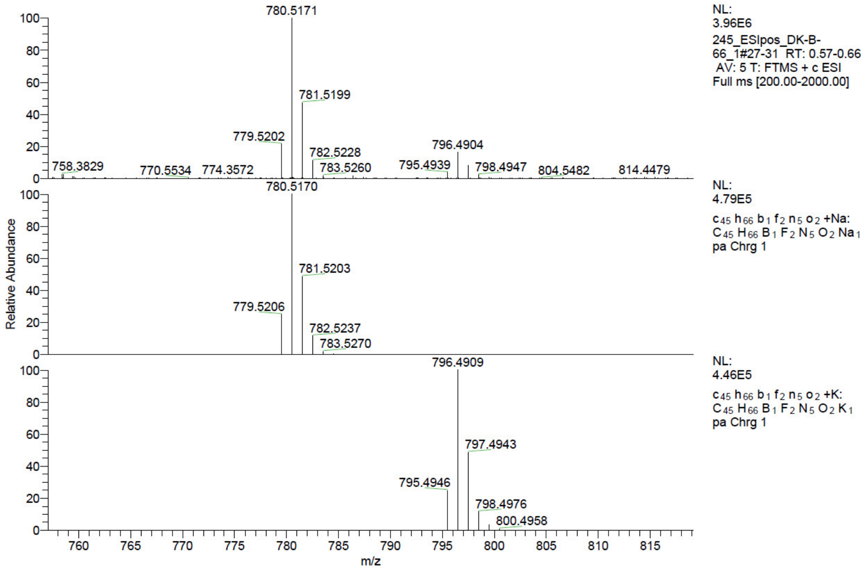

Figure S42. HRMS-ESI spectrum of **15.**

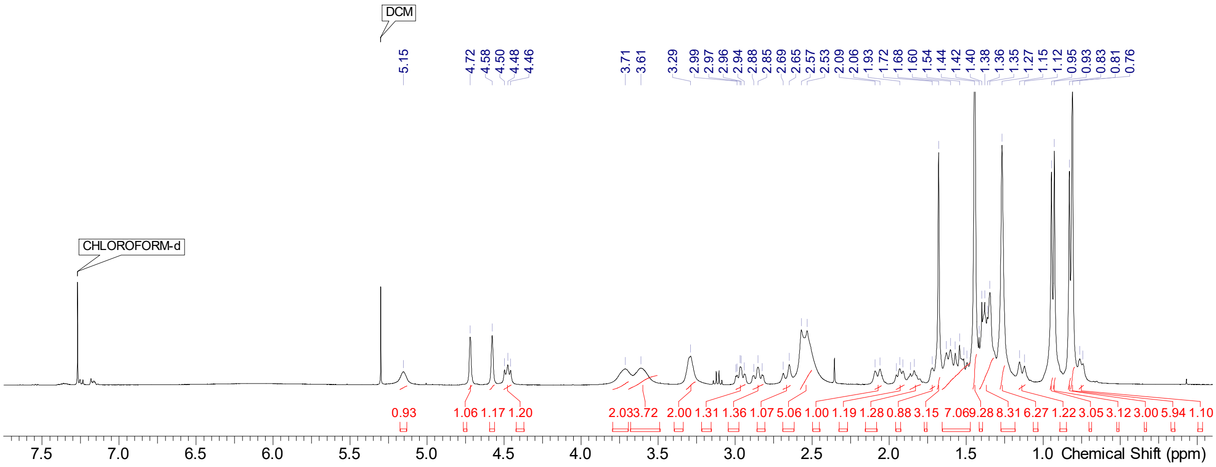

Figure S43. ^{1}H NMR of **16** in $CDCl_3$.

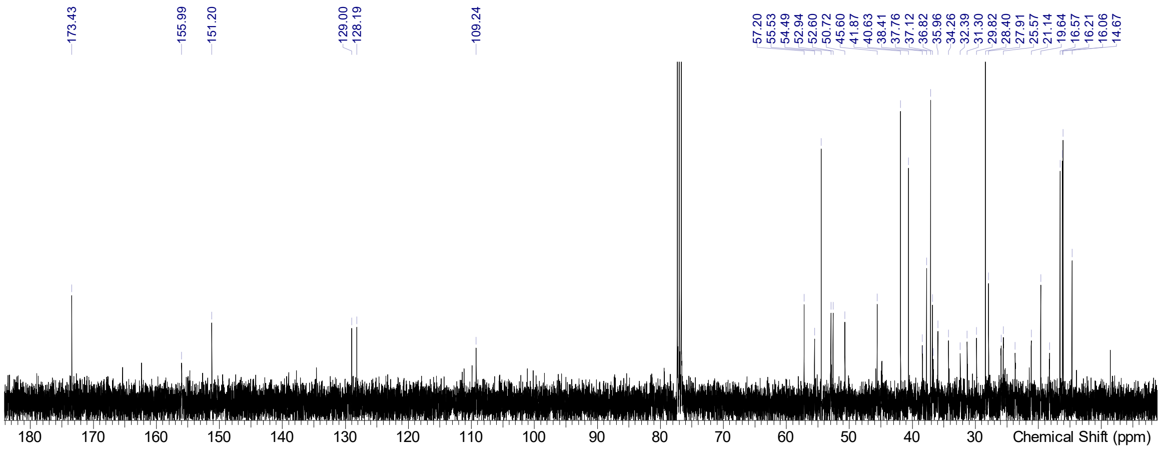

Figure S44. ^{13}C NMR of **16** in $CDCl_3$.

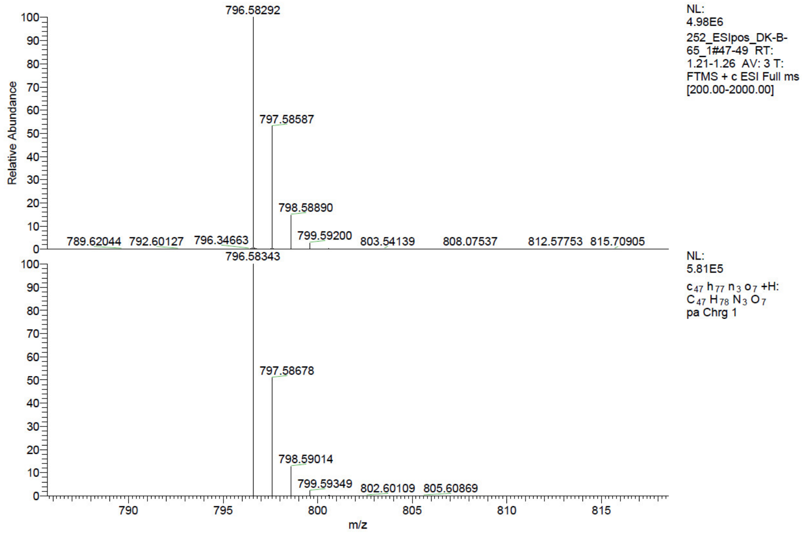

Figure S45. HRMS-ESI spectrum of **16.**

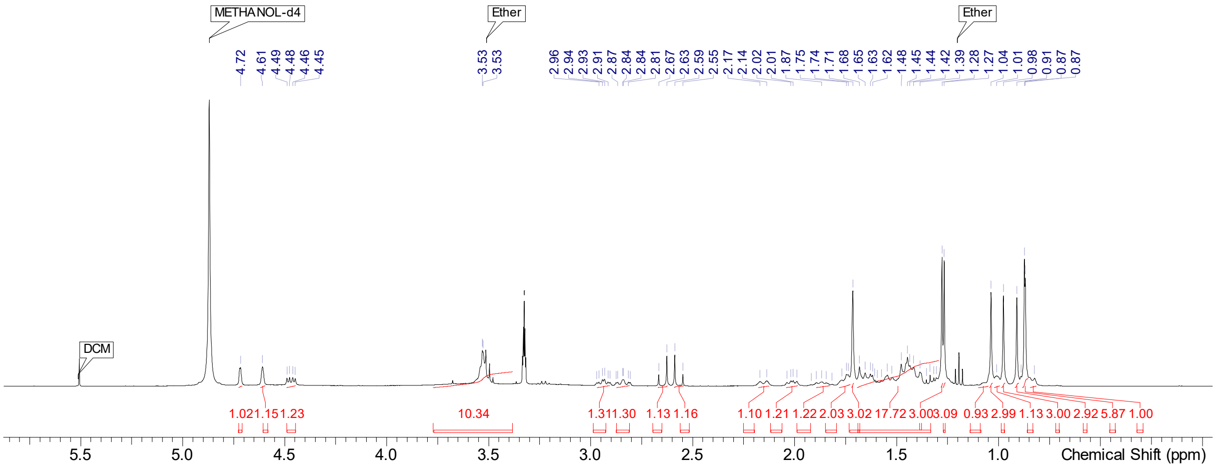

Figure S46. ^{1}H NMR of **17** in CD_3OD.

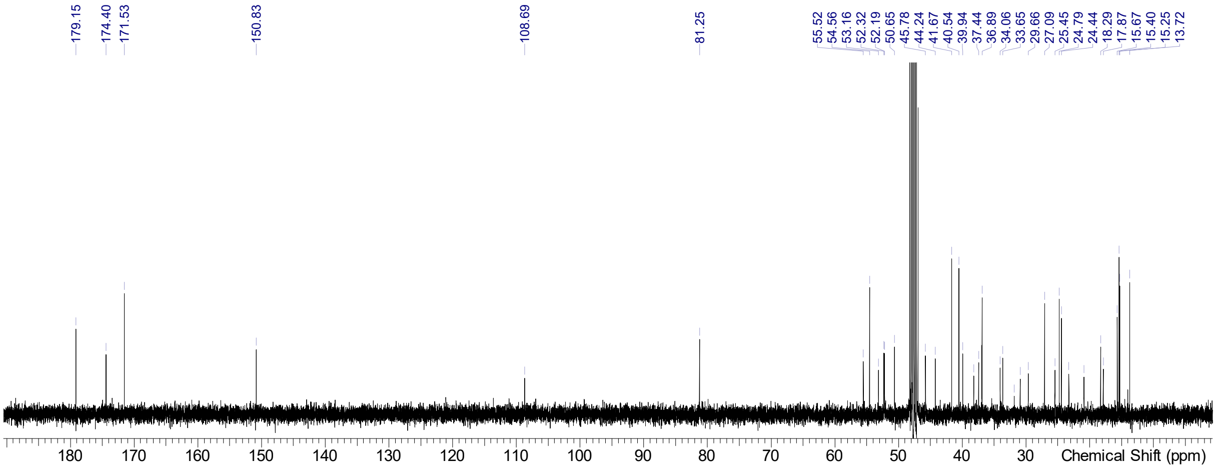

Figure S47. ^{13}C NMR of **17** in CD_3OD.

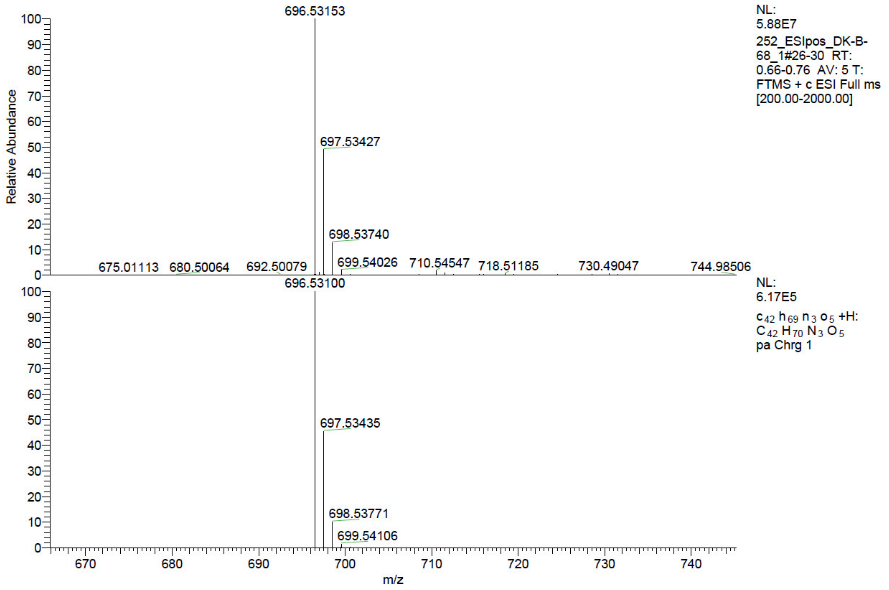

Figure S48. HRMS-ESI spectrum of **17.**

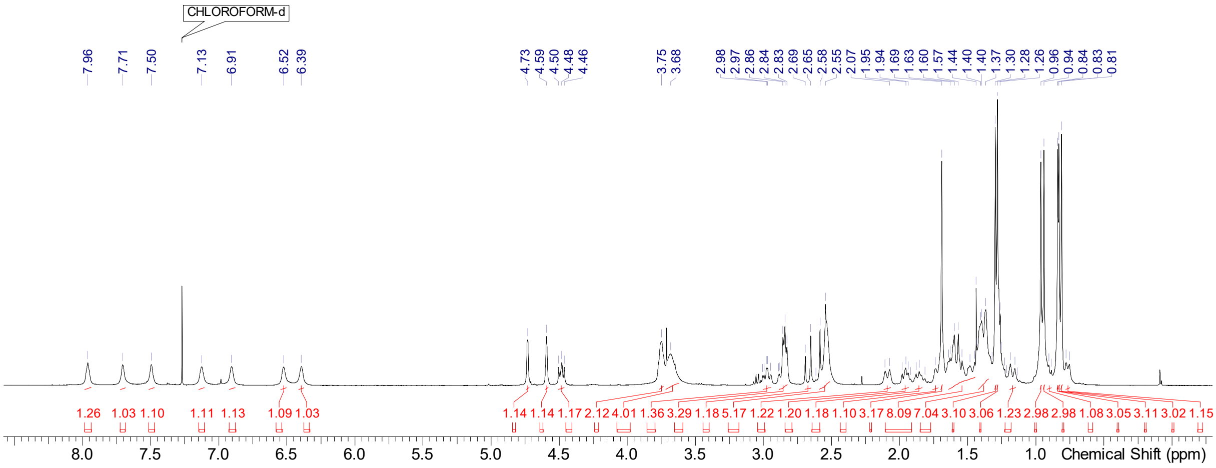

Figure S49. ^{1}H NMR of **18** in $CDCl_3$.

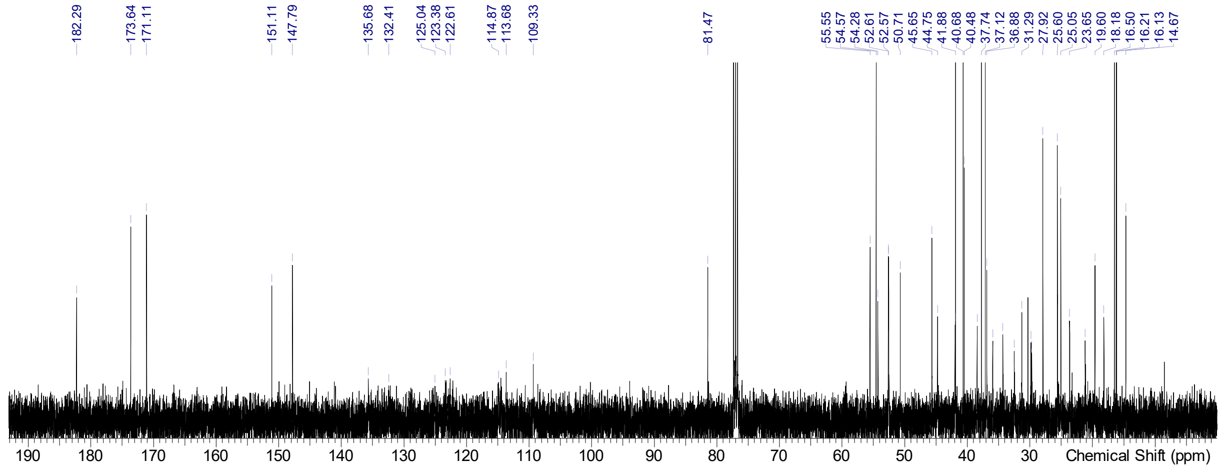

Figure S50. ^{13}C NMR of **18** in $CDCl_3$.

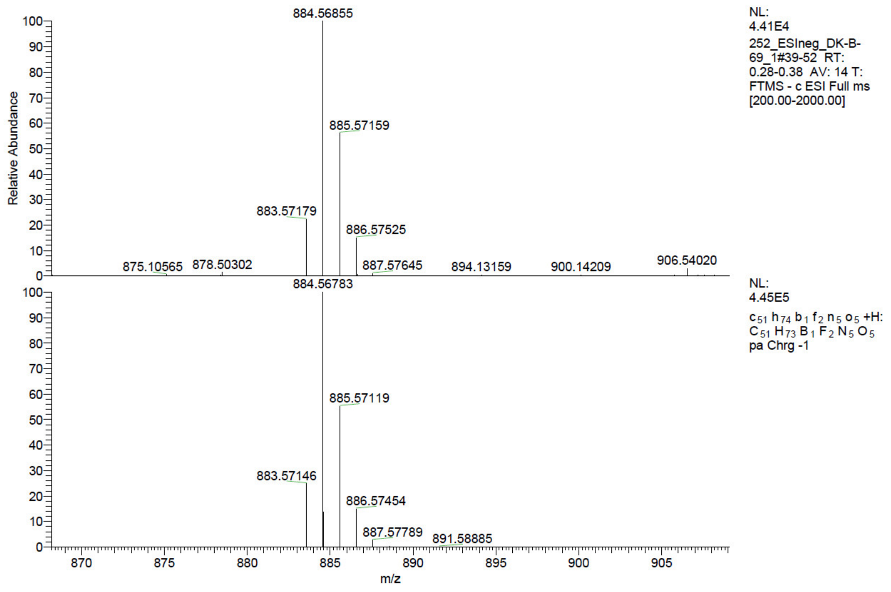

Figure S51. HRMS-ESI spectrum of **18.**

Table S1. Absorption maxima, emission maxima when excited at a wavelength of 380 nm, molar absorption coefficients and Stokes shifts of fluorescently labeled substances.

**Compd.**	$\lambda_{A, max}$ **(nm)**	$\lambda_{EM, max}$ **(nm)**	**ε (L·mol^{-1}·cm^{-1})**	**Stokes shift (nm)**
**4**	401	462	26 850	61
**5**	409	471	31 820	62
**6**	409	468	21 180	59
**10**	401	460	26 820	59
**15**	406	460	28 590	54
**18**	405	458	29 250	53

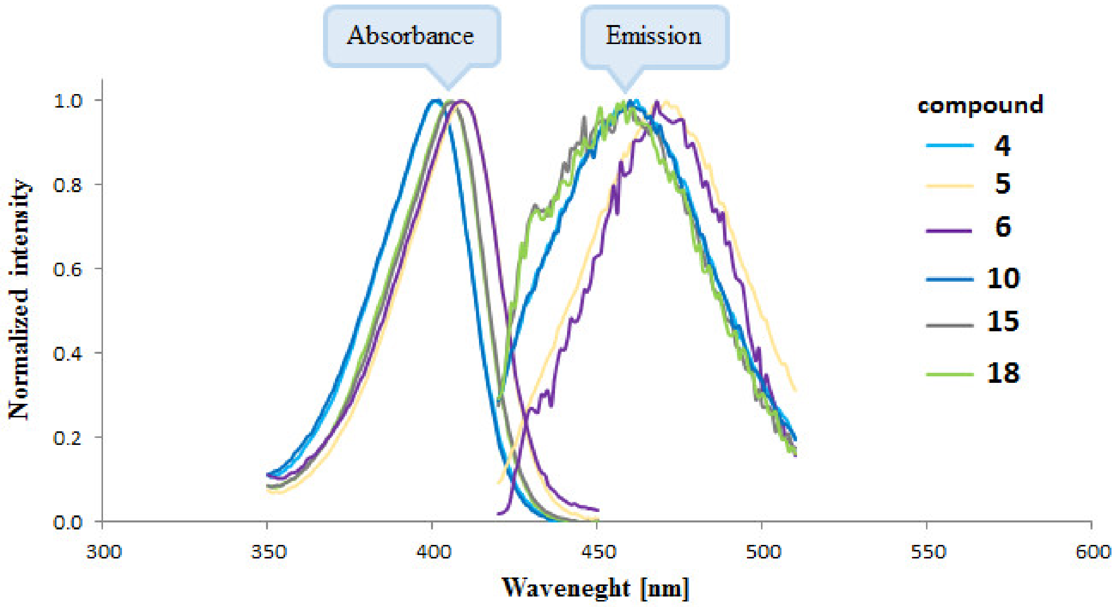

Figure S52. Normalized absorption and emission spectra of prepared fluorescent derivatives derived from BA.

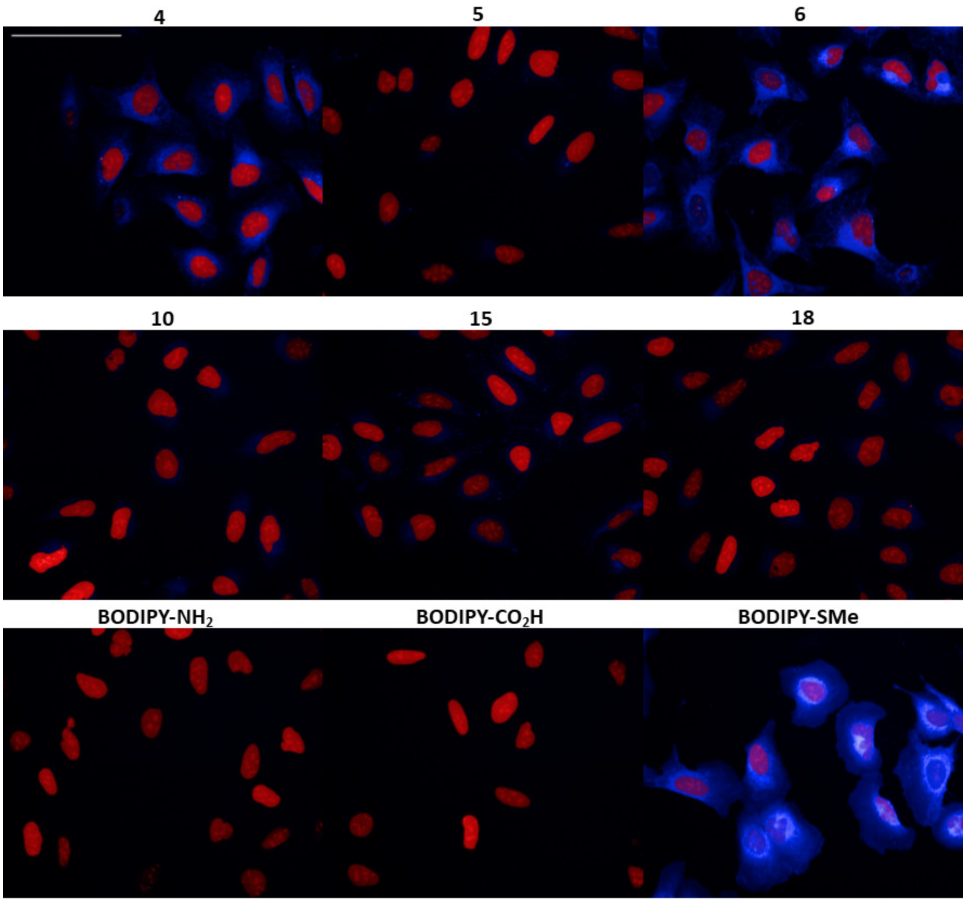

Figure S53. Pilot experiment with fluorescent derivatives. The size of the scale bar is 100 μm.

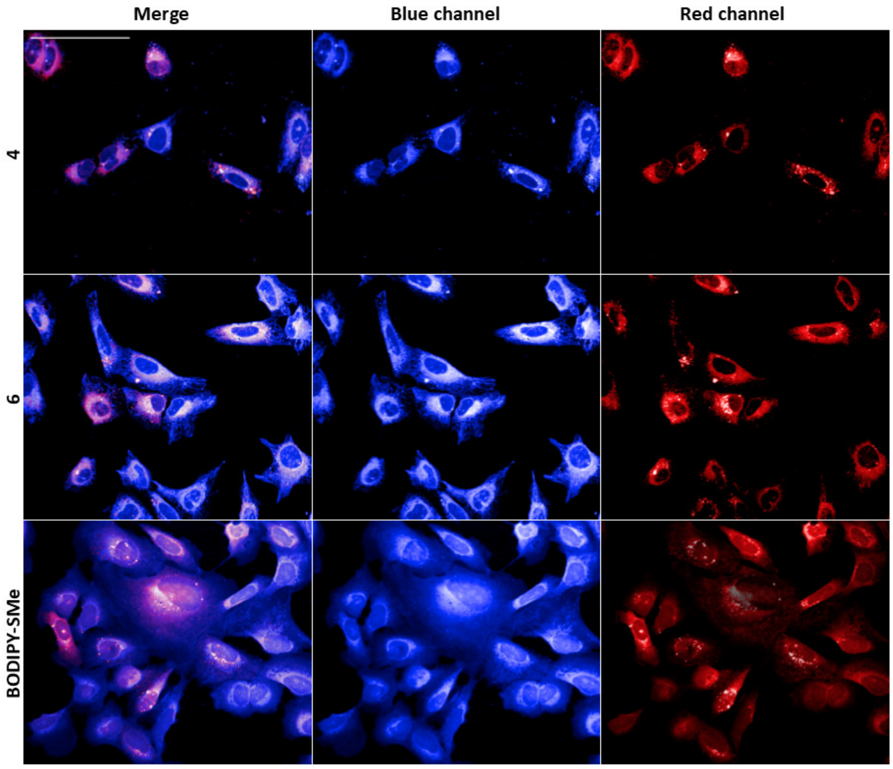

Figure S54. Images of U2OS-ER with entire microscopic fields. The size of the scale bar is 100 µm.

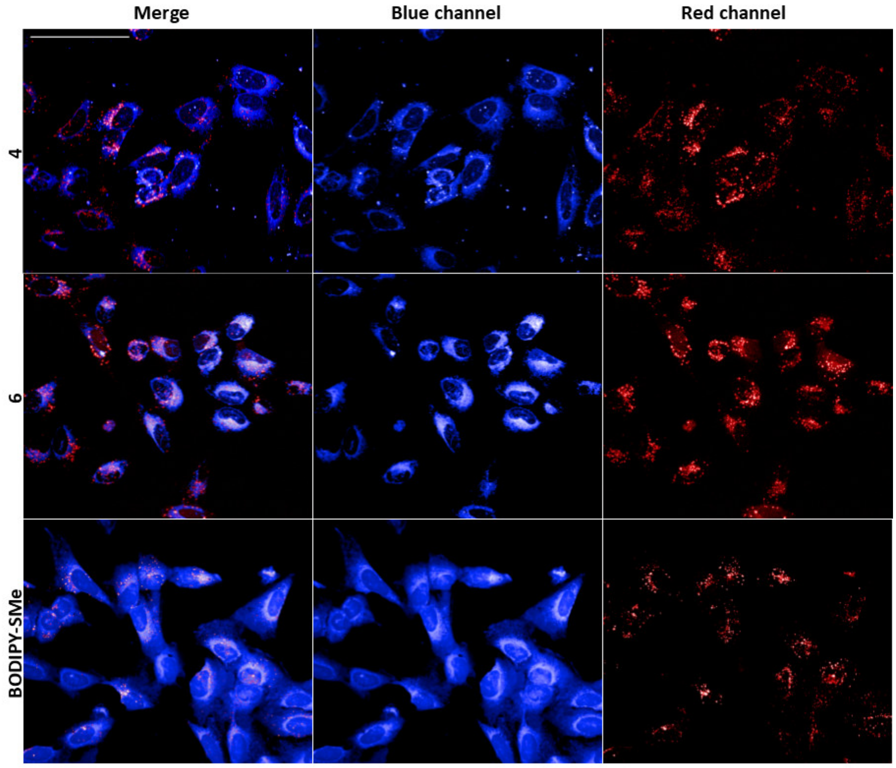

Figure S55. Images of U2OS-GA with entire microscopic fields. The size of the scale bar is 100 µm.

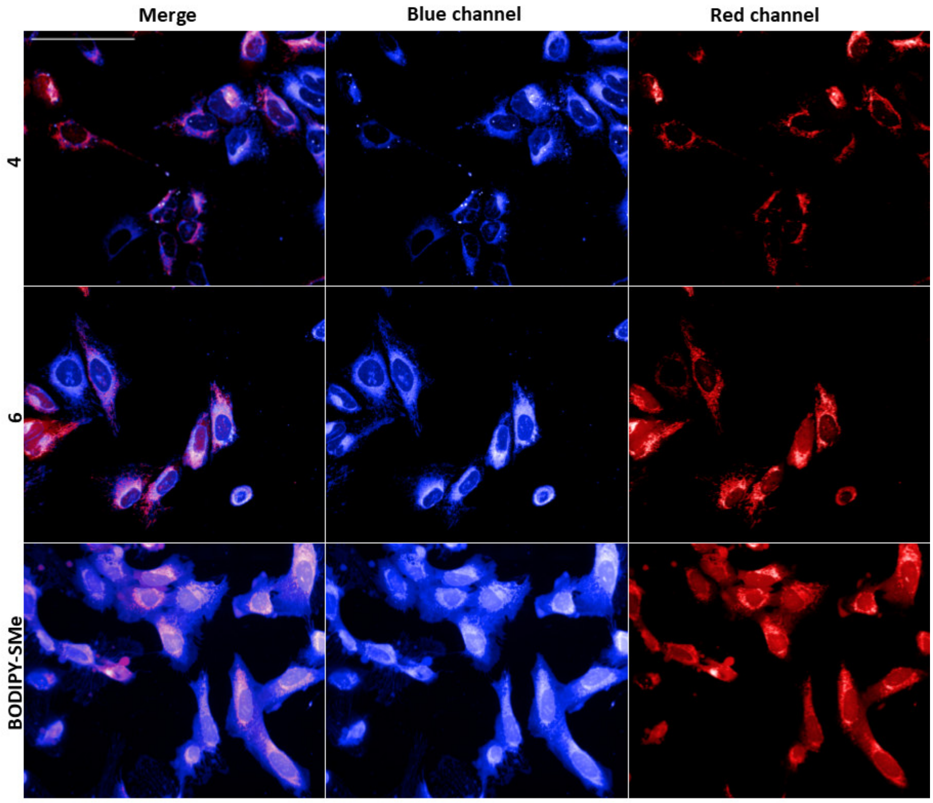

Figure S56. Images of U2OS-Mito with entire microscopic fields. The size of the scale bar is 100 μm.

Table S2. Calculated physical properties (pKa and logP) of the derivatives.

Compound	pKa*	pKa	pKa	LogP*
**BA**	4.61	15.19	n.a.**	7.85
**BT**	4.48	4.60	n.a.	9.26
**1**	15.17	15.82	n.a.	8.08
**2**	4.48	15.81	n.a.	9.30
**3**	15.17	16.00	n.a.	6.47
**4**	14.67	-	n.a.	6.27
**5**	n.a.	-	n.a.	7.41
**6**	9.74	-	n.a.	5.92
**7**	12.70	15.17	16.38	8.79
**8**	4.48	12.70	16.37	9.98
**9**	4.48	15.98	n.a.	8.06
**10**	4.48	n.a.	n.a.	7.15
**11**	4.61	n.a.	n.a.	8.82
**12**	12.74	n.a.	n.a.	9.90
**13**	12.74	15.17	n.a.	8.91
**14**	15.17	n.a.	n.a.	7.14
**15**	15.17	n.a.	n.a.	6.22
**16**	4.48	12.74	n.a.	10.32
**17**	4.48	n.a.	n.a.	8.39
**18**	4.48	n.a.	n.a.	6.96

*All values of pKa and logP (classic) were calculated with ACD/Percepta, Release 2021.1.0. For compounds **4**–**6** ACD/Percepta did not get reasonable value. The letter properties were calculated by PerkinElmer3D*Pro Version 20.0.0.41. For Compound **5**, PerkinElmer Chem3D* was not able to calculate reasonable value of pKa too. **n.a. not applicable.

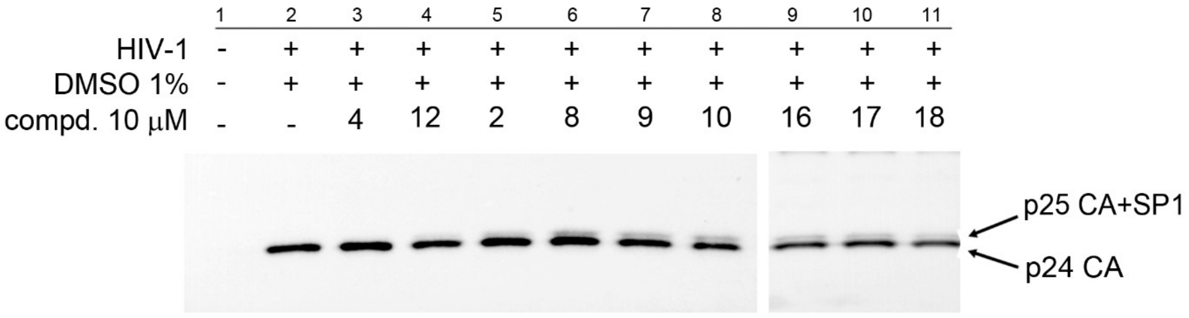

Figure S57. Effect of selected tested compounds on CA-SP1 processing of HIV-1 Gag polyprotein (a duplicate of western blot showed in Figure 5. in the article). HEK 293 cells produced HIV-1 particles pseudotyped with VSV-glycoproteins in the absence (lanes 2) or presence of selected tested compounds (lanes 3–11). At 48 h post-transfection, VSV-G pseudotyped HIV-1 viruses released from the HEK 293 cells were analyzed by western blot using an anti-HIV-1 CA antibody.
